# Mechanistic insights into the monotherapy and combination potential of FEN1 inhibition in cancer therapy

**DOI:** 10.1093/nar/gkaf1279

**Published:** 2025-12-08

**Authors:** Eeson Rajendra, Claudio A Lademann, Bethany Mason, Balca R Mardin, Lucy Armstrong, Silvia Peripolli, Julian Kreis, Timothea Konstantinou, Ulrich Pehl, Joerg Bomke, Stefanie Scharrelmann, David Perera, Birgitta Leuthner, Maria Filipa Pinto, Owen A Davis, Alessandro Galbiati, Ana Toste Rêgo, Claire L McWhirter, Elias Elinati, Ada Sala-Hojman, Julien Lefranc, Sam E Mann, Robert A Heald, Frank T Zenke, Graeme C M Smith, Helen M R Robinson, Lars T Burgdorf

**Affiliations:** Artios Pharma Ltd., B940, Babraham Research Campus, Cambridge CB22 3FH, United Kingdom; Merck Healthcare KGaA, Darmstadt 64293, Germany; Artios Pharma Ltd., B940, Babraham Research Campus, Cambridge CB22 3FH, United Kingdom; Merck Healthcare KGaA, Darmstadt 64293, Germany; Artios Pharma Ltd., B940, Babraham Research Campus, Cambridge CB22 3FH, United Kingdom; Artios Pharma Ltd., B940, Babraham Research Campus, Cambridge CB22 3FH, United Kingdom; Merck Healthcare KGaA, Darmstadt 64293, Germany; Artios Pharma Ltd., B940, Babraham Research Campus, Cambridge CB22 3FH, United Kingdom; Merck Healthcare KGaA, Darmstadt 64293, Germany; Merck Healthcare KGaA, Darmstadt 64293, Germany; Merck Healthcare KGaA, Darmstadt 64293, Germany; Artios Pharma Ltd., B940, Babraham Research Campus, Cambridge CB22 3FH, United Kingdom; Merck Healthcare KGaA, Darmstadt 64293, Germany; Artios Pharma Ltd., B940, Babraham Research Campus, Cambridge CB22 3FH, United Kingdom; Artios Pharma Ltd., B940, Babraham Research Campus, Cambridge CB22 3FH, United Kingdom; Artios Pharma Ltd., B940, Babraham Research Campus, Cambridge CB22 3FH, United Kingdom; Artios Pharma Ltd., B940, Babraham Research Campus, Cambridge CB22 3FH, United Kingdom; Artios Pharma Ltd., B940, Babraham Research Campus, Cambridge CB22 3FH, United Kingdom; Artios Pharma Ltd., B940, Babraham Research Campus, Cambridge CB22 3FH, United Kingdom; Merck Healthcare KGaA, Darmstadt 64293, Germany; Merck Healthcare KGaA, Darmstadt 64293, Germany; Artios Pharma Ltd., B940, Babraham Research Campus, Cambridge CB22 3FH, United Kingdom; Artios Pharma Ltd., B940, Babraham Research Campus, Cambridge CB22 3FH, United Kingdom; Merck Healthcare KGaA, Darmstadt 64293, Germany; Artios Pharma Ltd., B940, Babraham Research Campus, Cambridge CB22 3FH, United Kingdom; Artios Pharma Ltd., B940, Babraham Research Campus, Cambridge CB22 3FH, United Kingdom; Merck Healthcare KGaA, Darmstadt 64293, Germany

## Abstract

Flap endonuclease 1 (FEN1) is a structure-specific nuclease with critical functions in DNA replication and repair. FEN1 has been proposed as an anti-cancer drug target because of its synthetic lethal interaction with homologous recombination deficiency (HRD). However, the exploration of FEN1 in this context has been hampered by the lack of suitable small molecule tools. Here, we describe MSC778, a highly potent, specific and selective small molecule inhibitor of FEN1 nuclease activity. MSC778 directly engages FEN1 in cells, enhances its retention on chromatin, and selectively kills *BRCA*-deficient cells. Mechanistically, MSC778 suppresses DNA replication causing S-phase accumulation, DNA damage, and ultimately, cell death. Cancer cell panel screening identified Ewing sarcoma (EWS) cells as sensitive to MSC778, which is driven by the expression of *SLFN11*. In addition to HRD, CRISPR screening revealed a spectrum of synthetic lethal interactions between MSC778 and DNA damage response (DDR) factors, such as PARP1, USP1, PARG, and ATR. Furthermore, we demonstrate that combined inhibition of these factors with MSC778 induces synergistic killing of cancer cells. Together these data highlight FEN1 inhibition as an attractive precision oncology strategy either as monotherapy or as a combination therapy with a broad range of current and next generation DDR-targeting agents.

## Graphical Abstract

**Graphical Abstract:**
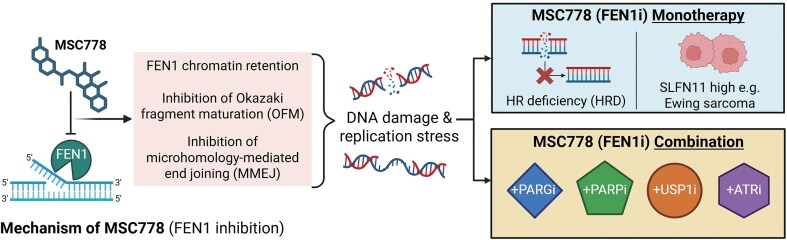

## Introduction

The structure-specific nuclease Flap endonuclease 1 (FEN1) is a member of the RAD2 nuclease family and its nucleolytic cleavage preference for 5′ flaps is essential for functions in DNA replication and repair [1, 2].

Faithful and processive DNA replication is achieved by continuous DNA synthesis by Polε on the leading strand and a complex discontinuous process on the lagging strand requiring the synthesis, nucleolytic processing and ligation of short nucleic acid stretches into one intact strand. During this highly coordinated process, Polα/primase synthesizes intermittent RNA–DNA primers that are subsequently extended by Polδ, into the adjacent primer, to yield contiguous stretches of unligated 5′-flapped Okazaki fragments (OF) [3, 4]. Okazaki fragment maturation (OFM) is the key step in generating the intact lagging strand and is primarily driven by FEN1-mediated cleavage of the RNA–DNA flap structures to generate a backbone nick, which is ligated by DNA Ligase 1 [5, 6]. OFM is essential for faithful DNA replication and defects in OF processing machinery, such as through defective FEN1 activity, lead to the accumulation of unligated OFs and single-stranded DNA (ssDNA) breaks (SSBs) that impact genome stability [7]. To circumvent this, SSB repair (SSBR) is initiated by recruitment of poly(ADP-ribose) polymerase 1 (PARP1), which mediates S-phase poly(ADP) ribosylation (PAR) that leads to SSBR platformed by XRCC1 [8]. Abrogation of SSBR by PARP1 inhibition triggers the accumulation and persistence of SSBs and ssDNA gaps that must undergo post-replicative repair to avoid turning into double-stranded breaks (DSBs), compromising cell survival [7, 9, 10]. There is also growing evidence, best characterized in the context of PARP inhibition, that excessive replication gap formation in itself may be a toxic lesion, without the need for conversion to a DSB for triggering apoptosis [11–14].

FEN1 also functions directly in multiple DNA repair mechanisms, resolving 5′ flaps during long-patch base excision repair (LP-BER) [15], the DSB repair (DSBR) pathway microhomology-mediated end joining (MMEJ) pathway [16], and DNA–protein crosslink (DPC) repair [17]. Genetically FEN1 has also been shown to be a synthetic lethal target in cells defective in machinery that mediates the DSBR pathway homologous recombination (HR), such as those driven by mutations in *BRCA1* and *BRCA2* [16, 18, 19].

Together, these data highlight FEN1 as a key enzyme in the DNA damage response (DDR) and a potential drug target through both synthetic lethal interactions and combination with agents targeting the DDR. To prosecute such a target for drug discovery, it is essential to have small molecule tools to interrogate the biology of FEN1, gain robust mechanistic insight into its function and identify clear strategies for disease positioning that define opportunities for monotherapy and combination opportunities. FEN1 inhibitors have been previously reported but they have shown limited potency and selectivity [20–23].

Here, we describe the *in vitro* characterization of MSC778, a novel small molecule inhibitor of FEN1 nuclease activity, with high potency and selectivity over other RAD2 family nucleases [24]. MSC778 binds to FEN1 in cells and enriches its recruitment to chromatin both in unchallenged cells, during unperturbed S-phase replication, and also to sites of DNA damage. Consistent with known genetics, FEN1 inhibition by MSC778 is synthetic lethal with HRD cells, driven by *BRCA* mutations. S-phase accumulation is hallmarked by defective OFM [substantially reduced DNA synthesis, induction of PARylation, and formation of replication protein A (RPA) foci marking ssDNA gaps], induction of DNA damage, and ultimately cell death. Expanding the potential target population for FEN1 inhibition, screening of cell line panels reveals a specific dependency of Ewing sarcoma (EWS) cells on FEN1 activity, driven by elevated expression of *SLFN11*, a transcriptional target of the *EWS–FLI* gene fusion, and a potential population biomarker for FEN1 inhibition. Mechanistically, *SLFN11* expression modifies the response to MSC778-mediated OFM, impacting replication without substantial ssDNA gap formation, marked by a lack of RPA foci formation. Genetic screening anchored to MSC778 not only confirms the synthetic lethality with DSBR/HR machinery but also reveals a robust synthetic lethality with the USP1/WDR48 deubiquitination complex. Furthermore, we validate and rationalize these mechanistic and phenotypic observations by showing that MSC778 can synergistically combine with pharmacological inhibition of PARP, PAR glycohydrolase (PARG), USP1, and ATR. Collectively these findings provide clear evidence that FEN1 constitutes a next generation DDR target and has defined monotherapy and combination potential in precision oncology.

## Materials and methods

### Biochemical assays

Detailed protocols are reported in an accompanying manuscript [24]. Brief protocols are below.

#### Assay ready plate generation

MSC778 was dissolved in dimethyl sulphoxide (DMSO) to give a 10 mM stock. FEN1/EXO1: compound was dispensed into black 384-shallow well microplates (Greiner #784076). The compound was dispensed as an 11-point dose response with a 3-fold serial dilution from a top concentration of 10 mM (to give a final in-assay concentration of 100 µM). GEN1/XPG: MSC778 was dispensed into black 1536-well plates (Greiner #782076). The compound was dispensed as a 20-point dose response with a 3-fold serial dilution from a top concentration of 10 mM (to give a final in-assay concentration of 100 µM). The volume of DMSO was adjusted to ensure that each well contained 1% (v/v) DMSO in the final assay. Control wells containing DMSO alone or inhibitor at sufficient concentration to cause complete inhibition of the reaction were also included.

#### DNA substrate preparation

Substrates were generated as a 20 µM stock by heating a mix of the appropriate oligonucleotides (Supplementary Table S1) diluted in annealing buffer (50 mM Tris pH 7.5, 3 mM MgCl_2_, and 100 mM NaCl to 65°C) for 5 min and cooling slowly to room temperature. Annealed substrates were stored as 20 µl aliquots at −80°C until required.

#### Enzyme activity assay

All assay buffers [FEN1: 20 mM Tris pH 7.5, 5 mM MgCl_2_, 100 mM KCl, 1 mM DTT, 0.01% IGEPAL CA-630, EXO1: 20 mM Tris pH 7.5, 5 mM MgCl2, 5% (v/v) glycerol, 1 mM DTT, 0.01% IGEPAL CA-630, GEN1: 20 mM Tris pH 7.5, 10 mM MgCl_2_, 10% (v/v) glycerol, 50 mM KCl, 1 mM DTT, 0.01% IGEPAL CA-630, XPG: 25 mM Tris pH 7.5, 5% (v/v) glycerol, 2 mM MgCl2, 20 mM KCl, 1 mM DTT, 0.01% IGEPAL CA-630, 0.1 mM EDTA] and “stop” reagents (20 mM Tris pH 7.5, 300 mM EDTA) were made fresh just prior to use. For each enzyme assay, the appropriate assay buffer was used for all intermediate dilutions.

A 2X stock of protein (20 pM FEN1, 1.6 nM EXO1, 1.6 nM GEN1, and 26 nM XPG) was made up in the appropriate assay buffer and 5 µl/well (FEN1/EXO1) or 3 µl/well (GEN1/XPG) was dispensed into assay plates that had been pre-dispensed with compound. The plates were covered and left to incubate at room temperature for 30 min before a 2× stock (80 nM) of the appropriate DNA substrate (Supplementary Table S1) was generated in the appropriate assay buffer, and 5 µl/well (FEN1/EXO1) or 3 µl/well (GEN1/XPG) was dispensed into the appropriate assay plates.

5 µl (FEN1/EXO1) or 3 µl per well (GEN1/XPG) of stop buffer (20 mM Tris pH 7.5, 300 mM EDTA) was added to all wells, then the plates were read on the CLARIOstar Plus (BMG LABTECH Ltd.) using the Fluorescein (FITC) wavelengths (Ex 483 ± 14, Em 530 ± 30, Dichroic auto/502.5) and auto focal height/gain settings.

#### Data analysis

All data analysis was carried out using Genedata Screener software. IC_50_ values were calculated using the SmartFit function.

### Bioinformatics

#### Gene expression data

RNA-seq data from 294 of the 299 broad indication cell line panel (Crown Bioscience) was generated on Illumina or MGIseq platforms and aligned using bowtie (version 0.12.9) using GRCh37 (Ensembl 66) as reference. No RNA data for HEPG2C3A, SW13, YCC1, SW156, and TTN were available. Gene expression levels were estimated using MMSEQ software [25], transformed to log2(FPKM), and finally transformed to TPM. Similarly, RNAseq data of the CCLE project were processed after downloading the data from the SRA database (accession number: PRJNA523380).

#### Biomarker analysis

For the gene expression biomarker analyses in the 299 cell line panel we identified genes involved in nuclease regulation, BER, the Fanconi Anemia pathway, MRE11–RAD50–NBS1 (MRN), HRD, replication stress (RS), drug efflux, drug metabolism, the ATR pathway, and genes from an internal DDR-associated gene set collection (unpublished). Together these comprised 1403 genes for biomarker analyses. TPM normalized gene expression values were subsequently log2 transformed and correlated with the relative area over the curve (relAOC) values, defined as the AOC divided by the sum of AOC and the area under the curve (AUC), from the dose–response assays, using Pearson’s correlation. Lastly, Benjamini–Hochberg *P*-value correction was applied to account for multiple testing [26].

For the homologous recombination deficiency (HRD) analysis in the breast cell line panel, HRD scores [27] were calculated on the basis of segmented copy number data of the DepMap molecular data release 23q2. Code was adapted from [28]. Subsequently, cell line responses were compared in HRD-low and HRD-high cell lines based on a HRD score cut-off of 42 [29].

Breast cell line panel responses have been compared in different disease subtypes as segmented according to [30].

### Cell culture

Cell lines used in this study are described in Supplementary Table S2 and were cultured under standard growth conditions (37°C, 5% CO_2_).

### Cell line generation

HeLa FEN1 SilenciX cells were transfected with pcDNA3.1(+)–FEN1–GFP (synthesis and cloning by GeneWiz) and individual colonies isolated under selection with 250 µg/ml Hygromycin B and 800 µg/ml G418 sulfate (Thermo Fisher Scientific). Stable expression of FEN1–GFP was confirmed by GFP detection by fluorescence microscopy and protein expression by western blot.

### Cell panel sensitivity screens

#### Monotherapy

Compound sensitization screens in a 93 cell line diverse indication panel and a 52 cell line specific breast cancer panel were performed at Oncolead. Cells were seeded at a density ensuring exponential growth for the duration of the experiment, treated for 5 or 7 days, respectively, fixed, stained with SRB, and quantified colorimetrically.

Compound sensitization screens in a large, broad indication 299 cancer cell line panel were performed at Crown Bioscience. Cells were seeded at a density ensuring exponential growth for the duration of the experiment, treated for 10 days, and proliferation measured using CellTiter-Glo (CTG).

GI_50_ (half maximal growth inhibitory concentration) values were calculated from the concentration response data and integrating pre-treatment control plates. In addition, the relative area above the curve (relAOC) was calculated.

#### Combination

Combination sensitization screens in 34 cancer cell lines (covering a range of indications) with a fixed dose (250 nM) of MSC778 versus 53 partner compounds (run in 5-point dose response) were performed at Oncolead. Cells were seeded at a density ensuring exponential growth for the duration of the experiment, treated for 5 days, fixed, stained with SRB, and quantified colorimetrically. Drug combination effects were calculated using the Bliss independence method [31]. Bliss synergy was calculated as the average delta of the observed effect *E*_OBS_ (i.e. the relative reduction of growth rate compared to untreated controls) and the calculated linear combination of the monotherapy treatment effects (E1 + 2 = E1 + E2 − E1 E2) for all concentrations used.


\begin{eqnarray*}
\textit{Blis}{s_{\textit{Score}}} = \frac{1}{n}\mathop \sum \limits_{i = 1}^n {E_{1 + {2_i}}} - {E_{OB{S_i}}}
\end{eqnarray*}

In this formulation, the resulting Bliss score is a continuous value between −1 and 1 and negative values indicate (potential) synergism, whereas positive values indicate (potential) antagonism.

### Cell viability assay (CTG)

Cells were seeded in 150 μl of media in 96-well plates or in 70 μl of media in 384-well plates pre-dosed with compounds (8–10 points dose response) with a D300e digital dispenser (Tecan) and incubated for 24 h at 37°C and 5% CO_2_. Cell proliferation at the end of the assay (7 days) was measured using CTG (Promega, G9243). CTG luminescence was normalized to vehicle treated control for each cell line, and data were fitted with a nonlinear regression curve (four parameters) to generate compound sensitivity curves and determine IC_50_ or EC_50_ (half maximal inhibitory/effective concentration) values as indicated.

For combination analysis, cells were seeded in 70 μl of media in 384-well plates pre-dosed with compounds (5-point dose–response matrix) with a D300e digital dispenser (Tecan). Cell proliferation was determined after 7 days using CTG. Analysis was performed with Genedata Screener (Compound Synergy Extension) to determine Synergy scores (Bliss model), using a formulation whereby the Bliss_excess_ is a continuous value and values higher than 2 are usually considered synergistic, and values between 0 and 2 are usually considered additive.

Commercially available compounds were used as received without further purification. Details are listed in Supplementary Table S3. Concentrations of compounds used in combination matrices are listed in Supplementary Table S4.

For assays requiring small interfering ribonucleic acid (siRNA) treatment, cells were seeded to six-well plates and 16–24 h later forward transfected with indicated siRNAs at 10 nM final concentration using Lipofectamine RNAiMAX (Invitrogen) according to manufacturer’s instructions. After 48 h, cells were washed with PBS, trypsinized, and reseeded for 4-day CTG viability assays or harvested for western blot analysis. siRNAs: siNTC = ON-TARGETplus Non-targeting Control Pool (D-001810-10-05, Horizon Discovery), siSLFN11#1 ON-TARGETplus Pool (L-016764-01-0005, Horizon Discovery), siSLFN11#2 (CAGGGAACCUUACGAAUUAdTdT [32], synthesized by Microsynth), and siSLFN11#3 (GGUAUUUCCUGAAGCCGAAdTdT [32], synthesized by Microsynth).

### Cell viability assay (Alamar Blue, AB)

Cells were seeded in 96-well plates and treated the day after seeding with a serial dilution of compounds in technical duplicates. After 7 days, viability was analyzed using the AlamarBlue reagent (BioRad). AlamarBlue was added in an amount equal to 10% of the volume in the well and after a 4–6 h incubation viability was determined by fluorescence analysis using a standard plate reader. Fluorescence was normalized to vehicle treated control for each cell line, and data were fitted in Genedata Screener to generate compound sensitivity curves and determine IC_50_ or EC_50_ (half maximal inhibitory/effective concentration) values as indicated.

### Chromatin retention assay

HeLa cells were seeded at a density of 4000 cells per well in 100 μl of media in collagen-coated 96-well plates (Revvity, #6055700). Sixteen to twenty-four hours after seeding, compounds were dispensed using the D300e digital dispenser (Tecan) to generate a multipoint dose–response curve, equalized to final DMSO concentration. After 24 h, cells were washed with phosphate buffered saline (PBS) and pre-extracted with 50 μl of ice-cold cytoskeletal (CSK) buffer [10 mM PIPES pH 6.8 (Sigma–Aldrich, P8203-50G), 100 mM NaCl (VWR, 27810.295), 300 mM sucrose (Sigma–Aldrich, S0389-500G), 1.5 mM MgCl_2_ (VWR, 8760.29), 5 mM EDTA (Sigma–Aldrich, E7889-100ML), 0.4% Triton X-1 (Sigma–Aldrich, T9285-500ML)], for 4 min on ice. Cells were then washed with PBS and fixed in 4% paraformaldehyde (PFA) (ChemCruz, sc-281692) for 10 min at room temperature.

For immunofluorescence staining, cells were washed with PBS then blocked and permeabilized with PBS containing 0.5% bovine serum albumin (BSA) and 0.5% Triton X-100 (blocking buffer) for 1 h at room temperature. Cells were then stained with the indicated markers (FEN1 or PARP1) by incubation with primary antibodies (Supplementary Table S5) diluted in blocking buffer, at room temperature for 4 h (FEN1) or overnight at 4°C (PARP1). Following three washes with PBS/0.1% Triton X-100 (PBS-TX), cells were incubated for 1 h at room temperature with secondary antibodies diluted 1:2000 in blocking buffer, that contained 4′,6-diamidino-2-phenylindole (DAPI 1:10 000, Thermo Fisher Scientific, #62248) to counterstain DNA. Cells were then washed three times with PBS-TX and then imaged in PBS using confocal microscopy (Operetta CLS, Revvity) using a long working distance ×20 objective, capturing 9 fields of view per well with 4 Z-slices and a Z-step of 1.2 μm.

### Colony formation assays

Exponentially growing DLD-1, DLD-1 BRCA2^KO^, SUM149PT, and MDA-MB-436 were seeded on 24-well plates at densities of 100, 400, 500, or 650 cells/well, respectively. Cells were treated with a serial dilution of FEN1 inhibitor and incubated for 11 days (DLD-1 and SUM149PT) or 14 days (DLD-1 BRCA2^KO^ and MDA-MB-436). Colonies were washed with PBS, fixed with 70% ethanol, and stained with 1% crystal violet solution (Sigma–Aldrich). Excess crystal violet solution was washed off using tap water and images were acquired using a GelCount Scanner (Oxford Optronix). Dye was solubilized with 10% acetic acid (VWR) and absorbance at 595 nm measured using a CLARIOstar plate reader (BMG LABTECH Ltd.). Raw absorbance data for each cell line were normalized to DMSO treatment. Data were fitted with a nonlinear regression curve (four parameters) to determine EC_50_ (half maximal inhibitory concentration) values.

### CRISPR screen

#### Focused library screen

The custom designed lentiviral library targeting 1100 genes was generated by Cellecta and used as described in [33]. Briefly, lentiviral particles were produced by co-transfecting 293FT cells with custom designed library (Cellecta), pMD2.G and psPAX2 plasmids using Lipofectamine 3000 (Thermo Fisher Scientific, L3000001) following manufacturer’s specifications. After 60 h, viral supernatant was collected and filtered through a 0.45 μm low protein binding membrane Steriflip HV/PVDF (Millipore), then concentrated 100× with Lenti-X Concentrator (Takara Bio, 631232).

Ten million hTERT RPE TP53^−/−^ iCas9 (doxycycline-inducible Cas9 expressing) cells per replicate, aliquoted to 600 000 cells per well of a six-well plate, were spinfected with the lentiviral library at 2000 rpm, for 2 h at 37°C, with the addition of 8 μg/ml polybrene (Sigma–Aldrich, TR-1003) to reach a multiplicity of infection of 0.3. Cells were left to recover in fresh medium for 24 h incubated at 37°C, 5% CO_2_. The infected cells were selected with 10 μg/ml puromycin (Thermo Fisher Scientific, A1113803) for 3 days. Following puromycin selection, Cas9 was induced with 1 μg/ml doxycycline (day 0). At day 7, cells were either treated with DMSO control or MSC778 (low dose, 500 nM or high dose, 2000 nM). Low dose treatment was repeated every three days for a total of four consecutive rounds. High dose treatment was applied once for 3 days and the medium was replaced with fresh medium after one round of treatment. Cells were collected at day 13 and day 20.

For preparation of the sequencing libraries, genomic DNA was isolated from the initial and final time points using QIAamp DNA Blood Kit (Qiagen, 51106) following manufacturer’s protocol. Eight micrograms of DNA was used as a template in two separate polymerase chain reaction (PCR) reactions of 50 µl for 12 cycles using plasmid-specific primers for the amplification of the single guide ribonucleic acid (sgRNA) coding sequences. An additional round of PCR amplification was run for 10 cycles for the addition of Illumina adapters and sample specific barcodes. Q5 NEBNext Hot Start polymerase master mix (M0543) was used for the PCR reactions according to manufacturer’s specifications. PCR reaction products of 390 bp were purified using 0.8× Agencourt AMPure XP (Beckman Coulter, A63880) and eluted in low EDTA TE buffer (Thermo Fisher Scientific, 12090015). All samples were pooled and sequenced using Illumina NextSeq500 platform (Illumina), using 75 base single reads.

#### Whole genome library screen

was performed similarly to the focused library screens. For this library 45 million hTERT RPE TP53^−/−^ iCas9 (doxycycline-inducible Cas9 expressing) cells were transduced, 300× coverage was kept at all times during the experiment, accordingly the cell numbers and the number of PCR reactions were adjusted.

The cell fitness is calculated based on the gRNA counts at the beginning and at the end of the experiment since the selection process may enrich or deplete sgRNAs from the cell population. Accordingly, the guide ribonucleic acids (gRNAs) that result in loss of cell fitness are expected to be depleted from the cell population at the final time point as compared to the first time point. To count the number of gRNAs in each library, and to perform downstream analyses we used three independent methods, DrugZ [34], MAGeCK [35], and Limma [36], which enable efficient analysis of genome-wide count-based screens on the initial and final timepoints. We classified hits as genes identified to be significantly depleted in 3/3 analysis methods. Reactome pathway enrichment was performed using the *clusterProfiler* package in R. Protein–protein interaction network analysis was performed using Cytoscape.

### CRISPR hit validation

To validate the hits from the CRISPR screen, independent gRNAs were used to target the hits, in an arrayed format using a solid-phase transfection method as previously described [37] with minor modifications.

#### Preparation of solid-phase transfection plate

A volume of 3 µl of Opti-MEM/sucrose solution (1.37% w/v) was combined with 2.5 µl RNAiMax (Thermo Fisher Scientific) and 3.5 µl of 1.5 µM multiguide sgRNA (Synthego), bringing the total volume to 11.5 µl with DNase-free water (Thermo Fisher Scientific). The mixture was briefly mixed and incubated at room temperature for 20 min. Following incubation, 7 µl of gelatin (Sigma–Aldrich #G9391) solution (0.2% w/v in DNase-free water) was added to the transfection mix, mixed briefly, and diluted 1:25 in RNA and DNase-free water. The final mix, designed for one reaction, was adjusted to the total volume needed for each gRNA (Supplementary Table S6) and the plates used, which were batch coated. For each gRNA, three wells were coated for DMSO treatment and three wells for MSC778 (500 nM) treatment, representing one replicate. A volume of 50 µl of the final mixture was dispensed into each well of a white, µCLEAR, TC-treated surface 96-well plate (Greiner #6550989). The plates were dried using a MiVac vacuum centrifuge (QUC-23050-B00) preheated to 60°C, then set to 37°C. After the drying process, plates were stored in a sealed box with silica desiccant for later use.

#### Seeding for the Hit validation

hTERT RPE TP53−/− iCas9 cells, which express doxycycline-inducible Cas9, were seeded at a density of 1000 cells per well onto the prepared solid-phase transfection plates. Treatment with 500 nM MSC778 or an equivalent volume of DMSO (Sigma–Aldrich #1.02950.0500) was administered on day 3 using a D300e digital dispenser (Tecan). Subsequently, on day 7, the cells were split at a ratio of 1:10 using TrypLE (Thermo Fisher Scientific) and retreated with 500 nM MSC778 or DMSO. On day 10, cell viability was evaluated using the CTG assay (Promega, G9243) according to the manufacturer’s instructions. The CTG luminescence was normalized to the DMSO-treated scramble gRNA control.

### Differential scanning fluorimetry 

A volume of 100 nl of each compound at stock concentration of 10 mM was dispensed into a 384-well skirted PCR Plate (MicroAmp^™^ Optical plate) using a D300e digital dispenser (Tecan). Wells were normalized such that each contained 1% (v/v) DMSO and final compound concentration of 100 µM.

FEN1 (1-380) FL-6His protein (1.25×) and protein 5× thermal shift dye (Thermo Fisher Scientific) were prepared separately in freshly prepared differential scanning fluorimetry (DSF) assay buffer (20 mM Tris–HCl pH 7.5, 100 mM potassium chloride, 5 mM magnesium chloride, and 1 mM dithiothreitol). Eight microliters of 1.25× Fen1 (10 µM) was added to the appropriate well, plate sealed with aluminium sealing film (Axygen), and centrifuged at 500 × *g* for 1 min before incubating for 30 min at 4°C. After the incubation period, the plate was centrifuged again at 500 × *g* for 1 min and 2 µl of 5× protein thermal shift dye was added. The plate was sealed with MicroAmp™ Optical Adhesive Film (Thermo Fisher Scientific) and centrifuged for another 1 min at 500 × *g*. The plate was then continuously heated 1.6°C/s to 25°C for 2 min after which a ramp of 0.05°C/s was applied up to 99°C over 2 min. Fluorescence was measured using a Quantstudio pro7.0 qPCR machine (Thermo Fisher Scientific) using internal optical ×1 filter (470 ± 15 nm) for excitation and m3 filter (586 ± 10 nm) for emission.

Each experiment was run in quadruplicates and melting temperatures (*T*_m_) calculated as the inflection point of the derivative function using the protein thermal shift software (Thermo Fisher Scientific). DeltaTm was calculated using the *T*_m_ of protein with 1% DMSO as reference.

### Flow cytometric analysis of apoptosis

Apoptosis was measured using annexin V and propidium iodide (PI) staining using an Annexin V-fluorescein isothiocyanate (FITC) apoptosis detection kit (Thermo Fisher Scientific, V13242) according to manufacturer’s instructions.

Briefly, cells were cultured in six-well plates and treated with FEN1 inhibitor at indicated concentrations for 72 h. The supernatant and adherent cells were collected and washed twice with ice-cold PBS, then re-suspended in 100 µl of 1× binding buffer. Annexin V-FITC conjugate (5 µl) and PI (1 µl) were added, and the cells were incubated at room temperature for 15 min in the dark. After addition of 300 µl of 1× binding buffer, the cells were analyzed using a flow cytometer (CytoFLEX, Beckman).

### Immunofluorescence

Cells were seeded at a density of 10 000 cells per well in 100 μl of media in collagen-coated 96-well plates (Revvity, #6055700). Twenty-four hours after seeding, cells were treated with the indicated doses of MSC2798778, MSC2798781, or DMSO (Apollo Scientific, BID1200) using a D300e digital dispenser (Tecan) for 24 h. Cells were washed with PBS and fixed in 4% PFA (ChemCruz, sc-281692) for 10 min at room temperature.

For immunofluorescence staining, cells were washed with PBS then blocked and permeabilized with PBS containing 0.5% BSA and 0.5% Triton X-100 (blocking buffer) for 45 min at room temperature. Cells were then stained with the indicated markers by incubation with primary antibodies (Supplementary Table S5) diluted in the blocking buffer, overnight at 4°C. Following three washes with PBS/0.1% Triton X-100 (PBS-TX), cells were incubated for 1 h at room temperature with secondary antibodies (Supplementary Table S5) diluted 1:2000 in blocking buffer that contained DAPI (1:10 000, Thermo Fisher Scientific, #62248) to counterstain DNA. Cells were then washed three times with PBS-TX and then imaged in PBS using confocal microscopy (Operetta CLS, Revvity) using a long working distance ×20 objective, capturing 7 fields of view per well with 5 Z-slices and a Z-step of 1.2 μm.

For total RPA, PCNA, and poly/mono-ADP ribosylation staining, prior to PFA fixation, cells were pre-extracted with 50 μl of ice-cold cytoskeletal buffer [10 mM PIPES pH 6.8 (Sigma–Aldrich, P8203-50G), 100 mM NaCl (VWR, 27810.295), 300 mM sucrose (Sigma–Aldrich, S0389-500G), 1.5 mM MgCl_2_ (VWR, 8760.29), 5 mM EDTA (Sigma–Aldrich, E7889-100ML), 0.5% Triton X-1 (Sigma–Aldrich, T9285-500ML), protease inhibitor tablet (Merck, 48679800), and phosphatase inhibitor tablet (Merck, 41659200)] for 4 min at room temperature.

For Poly/Mono-ADP Ribosylation staining, post PFA fixation, cells were permeabilized in ice-cold 100% methanol (VWR 20847.307) for 15 min at 4°C. Cells were then washed twice in PBS.

For EdU incorporation, cells in designated wells were labelled with 10 μM 5-ethynyl-2'-deoxyuridine (EdU) (Thermo Fisher Scientific, C10340) for 30 min prior PFA fixation. EdU incorporation was then detected using the Click-IT reaction according to the manufacturer’s instructions (Thermo Fisher Scientific, C10340), prior to immunofluorescence staining.

Imaging analysis was performed using Harmony 5.2. Maximum projection was used to merge Z-stacks into one image then nuclei were identified using DAPI staining.

1. Foci were quantified by setting up a contrast threshold higher than the background signal.
2. pKAP1 positive cells were selected using a mean intensity threshold.
3. Cell cycle analysis was performed by defining gates on a bivariate plot of DAPI total nuclear intensity and PCNA mean nuclear intensity. PCNA positive cells were scored as cells in S-phase. G1 and G2/M cells were scored as PCNA negative cells with differential DNA content.
4. PARylation and replication levels were quantified by measuring the mean nuclear fluorescence intensity, in PCNA positive cells, of poly/mono-ADP ribosylation and EdU, respectively.

### Laser microirradiation recruitment assay

HeLa FEN1 SilenciX (Tebu-Bio) + pcDNA3.1 FEN1–GFP cells were seeded at a density of 10 000 into 96-well plates (Revvity, #6055302) in DMEM/F-12 phenol red free media (Thermo Fisher Scientific, 21041025) supplemented with 10% FBS (PAN-Biotech, P30-3031). Cells were pre-sensitized with 10 μM 5-bromo-2’-deoxyuridine (BrdU, MedChemtronica, AB HY-15910) in media for an additional 24 h. Following treatment with either compound or vehicle control (DMSO) for 2 h at indicated concentrations, plates were mounted in an Olympus IXplore SpinSR spinning disk confocal microscope and cells were maintained at 5% CO_2_, and 37°C using a live-cell environmental chamber. Localized microirradiation damage was induced at a preselected region of interest (ROI), in an individual nucleus using the 405 nm laser (50% power, 5.1ms total dwell time, 25 repeats) and FEN1–GFP was imaged using the 488 nm laser (10% power, 300 ms exposure, binning 2 × 2) with a ×40 oil immersion objective (NA = 1.4). Images were taken before targeting and at 10 s intervals after targeting to monitor the recruitment of FEN1–GFP to the ROI over 6 min. The mean fluorescence intensity of FEN1–GFP in the damaged region was measured and divided by the intensity of the undamaged area in the same cell to assess recruitment kinetics using the CellTool software [38].

### MMEJ reporter assays

MMEJ resection-independent and MMEJ resection-dependent reporter assays performed as previously described [39]. In brief, HEK-293 cells were resuspended in supplemented SF nucleofection solution (Lonza) containing nanoluciferase DNA substrate and Firefly luciferase plasmid (Promega) at a ratio of 100 μl SF: 1500 ng nanoluciferase substrate: 1000 ng Firefly plasmid: ∼1.5 × 10^6^ cells. Cells were electroporated and recovered into fresh media before seeding into a 96-well plate pre-dosed with compounds and DMSO control.

After 24 h, Firefly and NanoLuc levels were detected using the Nano-Glo^®^ Dual-Luciferase^®^ Reporter Assay system (Promega) as per the manufacturer’s instructions. NanoLuc luminescence (reporter) was normalized to Firefly luminescence (control) to determine substrate repair. Percentage repair was calculated relative to the DMSO control.

### NanoBRET™ target engagement assay

The NanoBRET™ assay was set-up and performed by Promega, Madison, USA [40]. The tracer compound (“tracer 6”) was synthesized based on an in-house compound. A NanoLuc^®^-FEN1 expression plasmid was transiently transfected into HEK-293 cells and 20 µl of subsequent cell/DNA suspension dispensed into wells of 384-well assay plates. Tracer 6 (final concentration 125 nM) and test compounds (10 step 1:3 dilution from a top concentration of 10 µM) in 100% DMSO were dispensed with a Echo Labcyte (Beckman Coulter) in technical duplicates.

To induce NanoLuc activity, 10 µl of 3× detection substrate NanoBRET™ Nano-GLO^®^ Live Cell Solution in OptiMEM with digitonin (final concentration 150 µg/ml) were added per well. Kinetics were followed by luminescence multimode reader (DonorEm 450nm/AcceptorEm 610 nm) for 1 h. BRET ratios were calculated and resulting mBRET values were graphed versus compound concentrations and fitted to a nonlinear regression curve (four parameters) to determine IC_50_ values.

### Protein expression and purification

Detailed protocols are reported in an accompanying manuscript [24]. Briefly, protocols are below.

#### FEN1 (1-380)

Full-length FEN1 (1-380) was expressed in *Escherichia coli* and purified by affinity purification on a Nickel column followed by size-exclusion chromatography.

#### EXO1 (1-846)

Full length EXO1 was expressed and purified by Peak Proteins, Macclesfield, UK, as described previously [41].

#### GEN1 (1-527)

Truncated GEN1 (1-527) was expressed in insect cells and purified by affinity purification on a Streptrap column (Cytiva) followed by Heparin and size exclusion chromatography on a Superdex 200 column.

#### XPG (1*-1186*)

Full-length XPG (ERCC5) with a C-terminal Strep-tag was expressed in Sf9 insect cells and purified as described previously [42].

For all proteins, fractions were pooled and concentration determined by A280 nm measurement. Aliquots were snap frozen in liquid nitrogen and stored at −80°C for long term storage and until use.

### Subcellular fractionation

Cell fractions were prepared using Subcellular Protein Fractionation Kit for Cultured Cells (Thermo Fisher Scientific, 78840) following manufacturer’s instructions.

### Surface plasmon resonance

The kinetic parameters of the interaction between FEN1 and small molecules were assessed by surface plasmon resonance (SPR). FEN1(1-380)-6His was immobilized on a CM5 chip (Series S, Cytiva, Uppsala, Sweden) via standard amine coupling procedure at 25°C. Prior to immobilization, the carboxymethylated surface of the CM5 chip was activated with 400 mM 1-ethyl-3-(3-dimethylaminopropyl)-carbodiimide and 100 mM *N*-hydroxysuccinimide for 7 min. FEN1 was diluted to 10 µg/ml in 10 mM Bis–Tris pH 6.50 and injected in the presence of 2 µM of an active site binder on the CM5 chip for 3 or 4 min resulting in immobilization levels between 1300 and 3100 response units (RU). The remaining activated carboxymethylated groups were blocked with a 7 min injection of 1 M ethanolamine pH 8.50. HBS-N pH 7.40 was used as the background buffer during immobilization.

All compounds were prediluted in DMSO, diluted 1:50 in running buffer (20 mM Tris–HCl pH 7.50, 100 mM KCl, 1 mM DTT, 5 mM MgCl_2_, 0.05% Tween-20, and 2% DMSO) and injected at 10 different concentrations using two-fold dilution series. A DMSO solvent correction (10 steps, 1.4%–2.8%) was performed to account for variations in bulk signal and to achieve high-quality data. Interaction analysis cycles consisted of a 150 s sample injection (30 μl/min; association phase) followed by 300 s of buffer flow (dissociation phase).

Experiments were performed on a Biacore 8K+ (Cytiva, Uppsala, Sweden) at 25°C and the interactions were evaluated using the Biacore Insight evaluation software (version 5.0). All sensorgrams were processed by first subtracting the signal of the control surface (reference spot) from the binding response, followed by subtraction of the buffer blank injection from the reaction spot (double referencing). All datasets were fitted to a simple 1:1 Langmuir interaction model to determine the kinetic rate constants (using as offset RI = const = 0 and for Rmax either global or local, if the surface generation was not complete between consecutive cycles). Reported kinetic parameters are mean values from four independent FEN1 surfaces (*n* = 4).

### Synthesis of compounds

Commercially available reagents were used without purification and reactions were carried out under uncontrolled atmosphere unless otherwise stated. The typical mass spectrometer used for mass-directed HPLC was a Waters 3100 which detected masses between 100 and 700 g/mol. Full synthesis details will be provided in an accompanying manuscript.

### Western blot

Cells were washed in PBS, lysed directly in radio-immunoprecipitation assay buffer (RIPA) (Thermo Fisher Scientific, 89901) and the protein concentration of the extracts was quantitated using Bradford reagent (Bio–Rad 500-006) against a BSA standard curve. Extracts were made up in 4× NuPAGE LDS sample loading buffer (Invitrogen, NP0008) supplemented with 100 mM dithiothreitol (Sigma–Aldrich 43816-10ML), and incubated at 70°C for 10 min. Lysates (20–30 µg) were resolved by sodium dodecyl-sulphate (SDS)–polyacrylamide gel electrophoresis (SDS–PAGE) on NuPAGE 3%–8% tris-acetate gels (Invitrogen, STM4006) in NuPAGE tris–acetate running buffer (Invitrogen, LA0041), or NuPAGE 4%–12% bis–tris gels (Invitrogen, WG1402BX10) in NuPAGE MOPS SDS running buffer (Invitrogen, NP0001). Bis–tris gels were transferred onto nitrocellulose using an iBlot system (Invitrogen) for 10 min at 20 V. Tris–acetate gels were wet-transferred in 1× NuPAGE Transfer Buffer (Invitrogen, NP0006-1) supplemented with 10% ethanol to nitrocellulose membranes (Amersham, GE10600002). 5% BSA/Tris-buffered saline + 0.01% Tween-20 (TBST) or 5% milk/TBST was used for blocking and incubation steps. Membranes were probed overnight at 4°C with indicated primary antibodies. The membrane was washed thrice for 5 min with TBST and incubated with fluorescent dye-conjugated secondary antibodies for 1 h at room temperature. After four 5 min washes with TBST, fluorescent signals were detected on a LI-COR Odyssey M Imager. Antibodies used in this study are described in Supplementary Table S5.

### Software

Software used for data collation, analysis and representation are listed in Supplementary Table S7.

### Statistical analysis

Data were collated in Microsoft Excel and graph generation, curve fitting and statistical analyses were performed in GraphPad Prism. Statistical significance was determined as indicated in the figure legends; “n.s” = not significant (*P* > 0.05), *= *P* ≤ 0.05, **= *P* ≤ 0.01, ***= *P* ≤ 0.001, ****= *P* ≤ 0.0001.

## Results

### Discovery of MSC778, a potent and selective small molecule FEN1 inhibitor

**Figure 1.**
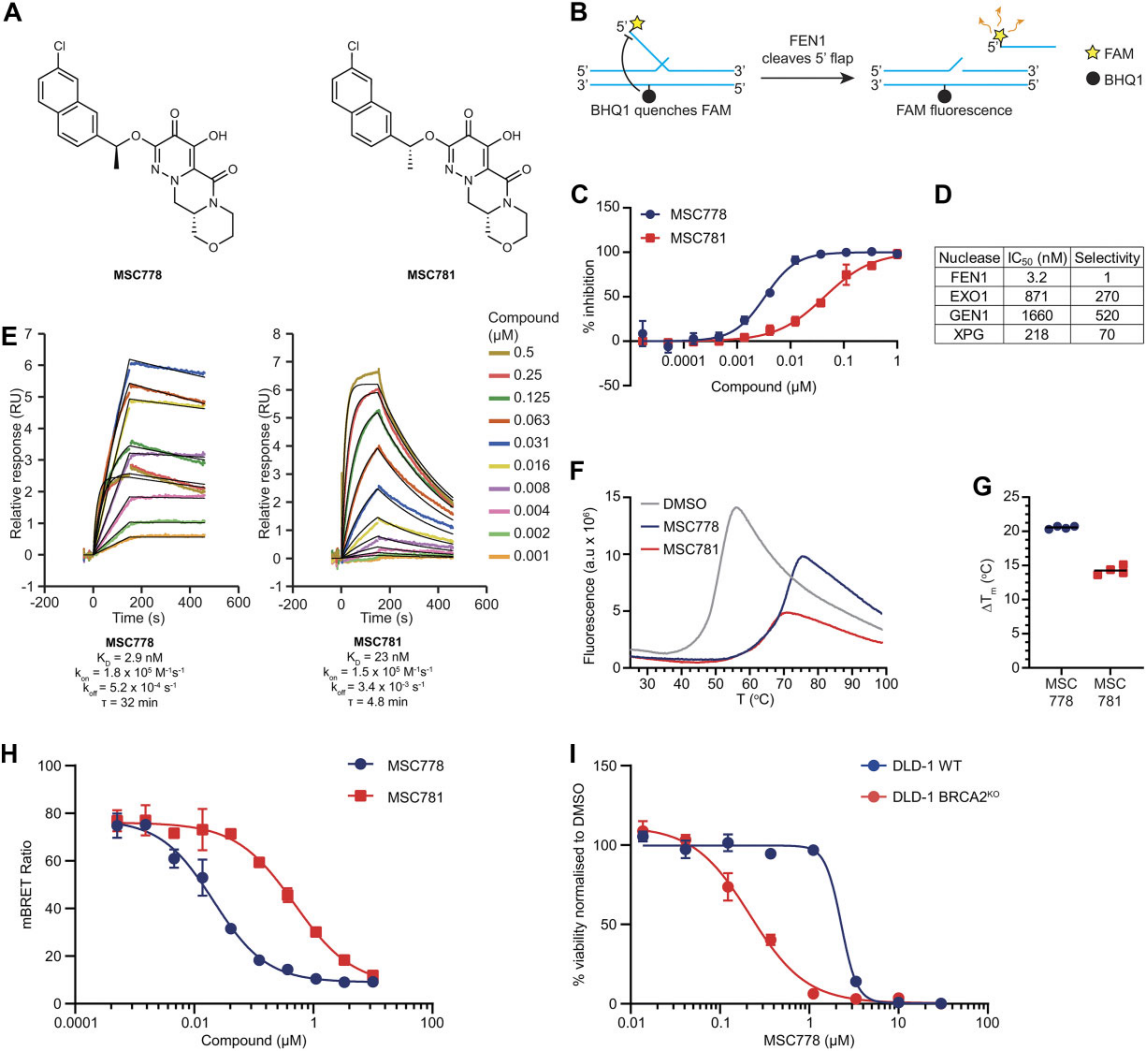
Characterization of FEN1 inhibitor MSC778 (**A**). Structures of FEN1 inhibitor MSC778 and diastereomer MSC781. (**B**) Schematic of the biochemical assay measuring FEN1-dependent cleavage of a 5′ flap substrate. (**C**). Inhibition of FEN1 by MSC778 and MSC781 in the biochemical assay outlined in (B). Data represent the mean ± SD from three biological replicates, each performed in technical singlicate. (**D**) 778 is highly selective for FEN1 inhibition over other RAD2 nuclease family members. IC_50_ values were determined for indicated nucleases: FEN1 (*n* = 3, SD = 0.14), EXO1 (*n* = 3, SD = 223), GEN1 (*n* = 11, SD = 569), XPG (*n* = 10, SD = 0.86) against cognate substrates (Supplementary Table S1). Selectivity was calculated as (nuclease IC_50_)/(FEN1 IC_50_) and rounded. (**E**) Representative SPR dose responses obtained by injection of 778 and 781 over FEN1(1-380)-6His immobilized on a CM5 chip. Double-referenced data (colored traces) were fit to a 1:1 Langmuir interaction model (black traces). Reported kinetic parameters are mean values from four independent FEN1 surfaces (*n* = 4). (**F**) Representative fluorescence thermal spectra for FEN1 in the presence of reference 1% DMSO (gray), 778 (blue), or 781 (red). (**G**) Mean difference in melting temperature (ΔTm) for the 778 (blue) or 781 (red) normalized to the DMSO reference. Data represent the mean of technical quadruplicates. (**H**) NanoBRET™ cellular target engagement assay. Data represent the mean ± SD from two biological replicates, each performed in technical duplicates. (**I**) Cell viability assays (AlamarBlue) were performed in DLD-1 and DLD-1 BRCA2^KO^ cells after 7 days treatment with MSC778. Data represent mean ± SEM of three biological replicates, each performed in technical duplicates. Corresponding data with MSC781 are shown in Supplementary Fig. S1B.

A bespoke metal-chelating fragment library was screened to identify inhibitors of FEN1 nuclease activity. Hits were optimized to improve potency and physicochemical properties to give MSC778 [24] and its diastereomer MSC781, which served as a control to demonstrate the specificity of FEN1-driven phenotypes (Fig. 1A).

A fluorescence-based 5′-flap cleavage assay, in which quenching of the fluorescence of a fluorescein amidite (FAM) moiety is alleviated by nucleolytic cleavage, was used to measure the nuclease activity of FEN1 (Fig. 1B and Supplementary Fig. S1A). MSC778 robustly inhibited FEN1 nuclease activity and was 16-fold more potent than MSC781 (Fig. 1C). Importantly, MSC778 did not inhibit other RAD2 family nucleases and is thus highly selective for FEN1 (Fig. 1D and Supplementary Fig. S1A) [2, 24, 43]. Real-time kinetic studies of inhibitor binding to apo FEN1 were performed using SPR analysis. Although both compounds had similar association rate constants (*k*_on_), MSC778 had a slower dissociation rate constant (*k*_off_) than MSC781 and therefore a longer residence time on FEN1 (Fig. 1E and Supplementary Fig. S1A). Confirming direct binding, DSF showed that MSC778 thermally stabilized FEN1, and this was to a greater extent than MSC781 (Fig. 1F and G, and Supplementary Fig. S1A).

Having demonstrated direct binding to FEN1 through biophysical means, we next confirmed target engagement in cells using a Nanoluciferase-based bioluminescence resonance energy transfer (NanoBRET™) assay [40]. In this assay, NanoBRET occurs between a fluorescent moiety (acceptor) on a tracer molecule and the NanoLuc tag (donor) on FEN1 that are in close proximity. Competitive displacement of the tracer molecule by MSC778 reduced the BRET signal between the tracer and FEN1 (EC_50_ 22 nM, *N* = 2) in a concentration-dependent manner. Aligning to the split in potency observed biochemically (Fig. 1C), MSC778 was 23-fold more potent than MSC781 (Fig. 1H).

Genetic ablation of FEN1 is synthetic lethal with *BRCA2* inactivation [16]. Confirming on-target phenotypic activity driven by pharmacological inhibition of FEN1, DLD-1 *BRCA2*^KO^ cells were ∼10-fold more sensitive than isogenic parental DLD-1 wild-type (DLD-1) cells to MSC778 (EC_50_ 0.22 µM versus 2.3 µM, *N* = 3) (Fig. 1I). Both cell lines were much less sensitive to MSC781 (Supplementary Fig. S1B).

**Figure 2.**
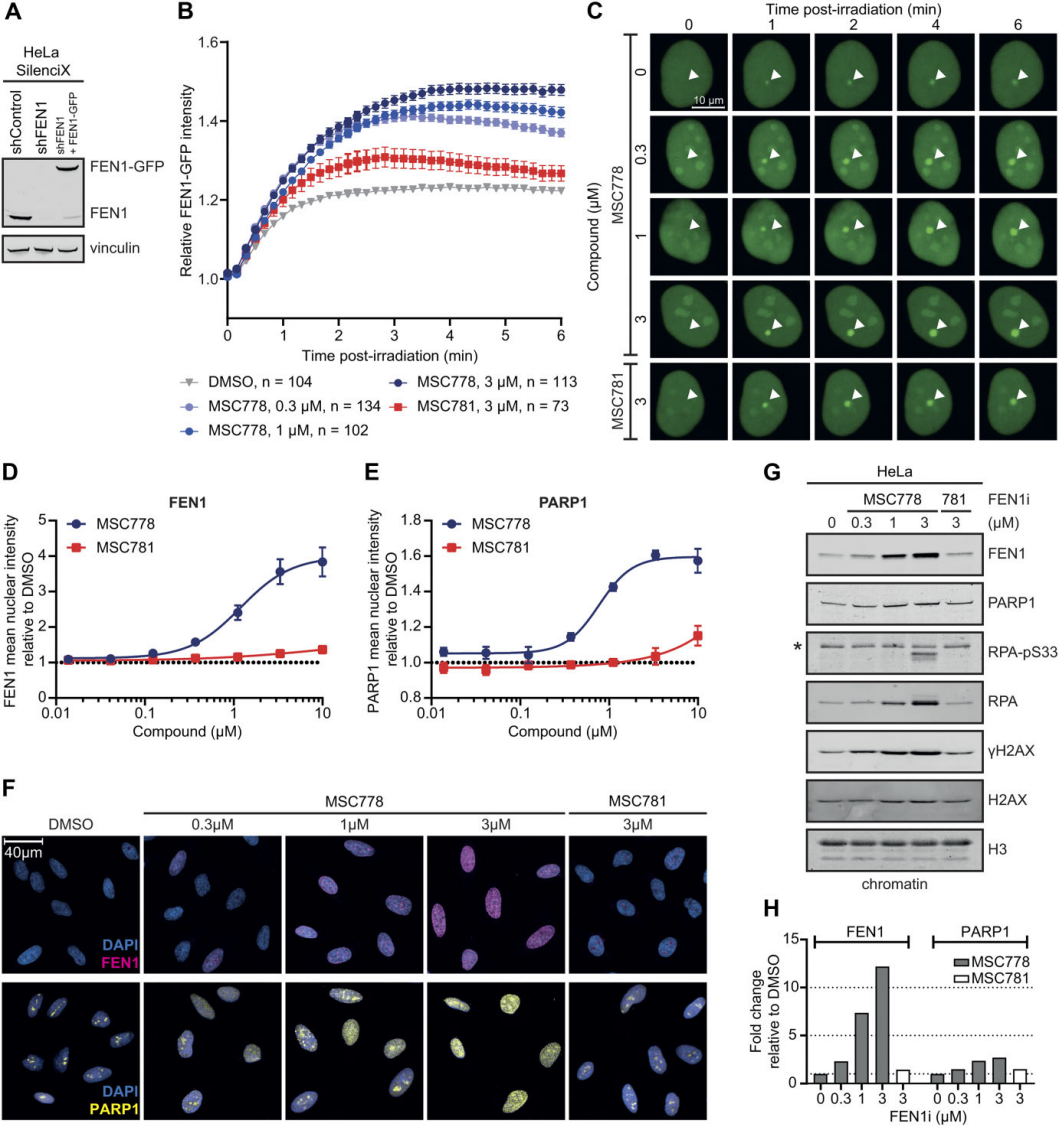
MSC778 increases recruitment of FEN1 to sites of DNA damage and enriches its chromatin retention in cells (**A**). Western blot of FEN1 in HeLa SilenciX cells. (**B**) Kinetics of recruitment of FEN1–GFP to sites of laser microirradiation upon treatment with MSC778 or MSC781. Data represent mean ± SEM of the indicated number of cells for each concentration of indicated compounds over three biological replicates. (**C**) Representative images of a time course of FEN1–GFP recruitment to damaged ROI upon treatment with indicated compounds. White arrow indicates microirradiation site. (**D**) Chromatin retention of endogenous FEN1 was assessed by immunofluorescence imaging of CSK-extracted HeLa cells after 24 h treatment with FEN1 inhibitor. Data represent mean ± SEM of three biological replicates each performed in technical triplicates. For each replicate sample *n* > 600 cells were scored per condition. (**E**) Chromatin retention of endogenous PARP1 was assessed by immunofluorescence imaging of CSK-extracted HeLa cells after 24 h treatment with FEN1 inhibitor. Data represent mean ± SEM of three biological replicates each performed in technical triplicates. For each replicate sample *n* > 350 cells were scored per condition. (**F**) Representative images of cells imaged in panels (D and E). (**G**) Chromatin enrichment of endogenous FEN1 was assessed by western blot of FEN1 in chromatin extracts upon subcellular fractionation of HeLa cells after 24 h treatment with FEN1 inhibitor. “*” is a nonspecific band. Full western blot is shown in Supplementary Fig. S2A. (**H**) Western blot quantification. Data represent the signal intensity relative to H3 normalized to DMSO control. Additional quantification is shown in Supplementary Fig. S2B.

We next interrogated how MSC778 modulated the function of FEN1 in cells. Commensurate with its functions in DNA repair, FEN1 has been shown to be recruited to sites of DNA damage generated by laser microirradiation [44]. Using a cell line in which endogenous FEN1 expression is suppressed and a FEN1 species C-terminally tagged with GFP is stably overexpressed (Fig. 2A), the recruitment of FEN1–GFP to damage was monitored by live cell imaging. MSC778 titratably increased the recruitment and retention of FEN1–GFP to DNA damage, more so than MSC781 (Fig. 2B and C). These data suggested that MSC778 might elicit a chromatin “trapping”-like mode of action on FEN1. To further investigate this, we assessed the chromatin retention of endogenous FEN1 in unchallenged HeLa cells. Indirect immunofluorescence assays revealed a clear increase in FEN1 chromatin enrichment (EC_50_ 1.2 µM), which was associated with a concomitant increase in SSB sensor PARP1 (Fig. 2D–F). Western blot analysis confirmed these observations and highlighted the induction of replication stress (RS) [45, 46], marked by RPA pS33 (pRPA) [47], and DNA damage, marked by H2AX pS139 (γH2AX) [48] (Fig. 2G and H, and Supplementary Fig. S2A and B) [45]. Chromatin enrichment (of either FEN1 or PARP1) and DNA damage signalling were not enhanced upon treatment with MSC781. Together, these data suggest that MSC778 enhances the recruitment of FEN1 to chromatin and that inhibition additionally triggers PARP recruitment to chromatin. As FEN1 nuclease activity is inhibited, we speculate that FEN1 cannot process DNA substrates, leading to its retention on chromatin, elevated RS and DNA damage, in accordance with the known role of FEN1 in DNA replication.

FEN1 has also been proposed to function in MMEJ [16] where 5′-flap cleavage is essential to remove the flapped DNA strand displaced by Polθ-mediated polymerization. The function in this “backup” DSBR pathway may also contribute to the synthetic lethality of FEN1 in *BRCA*-mutant HRD cells. Using extrachromosomal MMEJ reporter assays [39], MSC778, but not MSC781, inhibited MMEJ (EC_50_ 1.0 µM and 0.84 µM in resection independent and dependent reporters, respectively) in agreement with previous reports, but with 10–20-fold lower potency than ART558, an inhibitor of the polymerase domain of Polθ (Supplementary Fig. S2C and D) [39, 49].

### *BRCA* mutation sensitizes cells to MSC778

**Figure 3.**
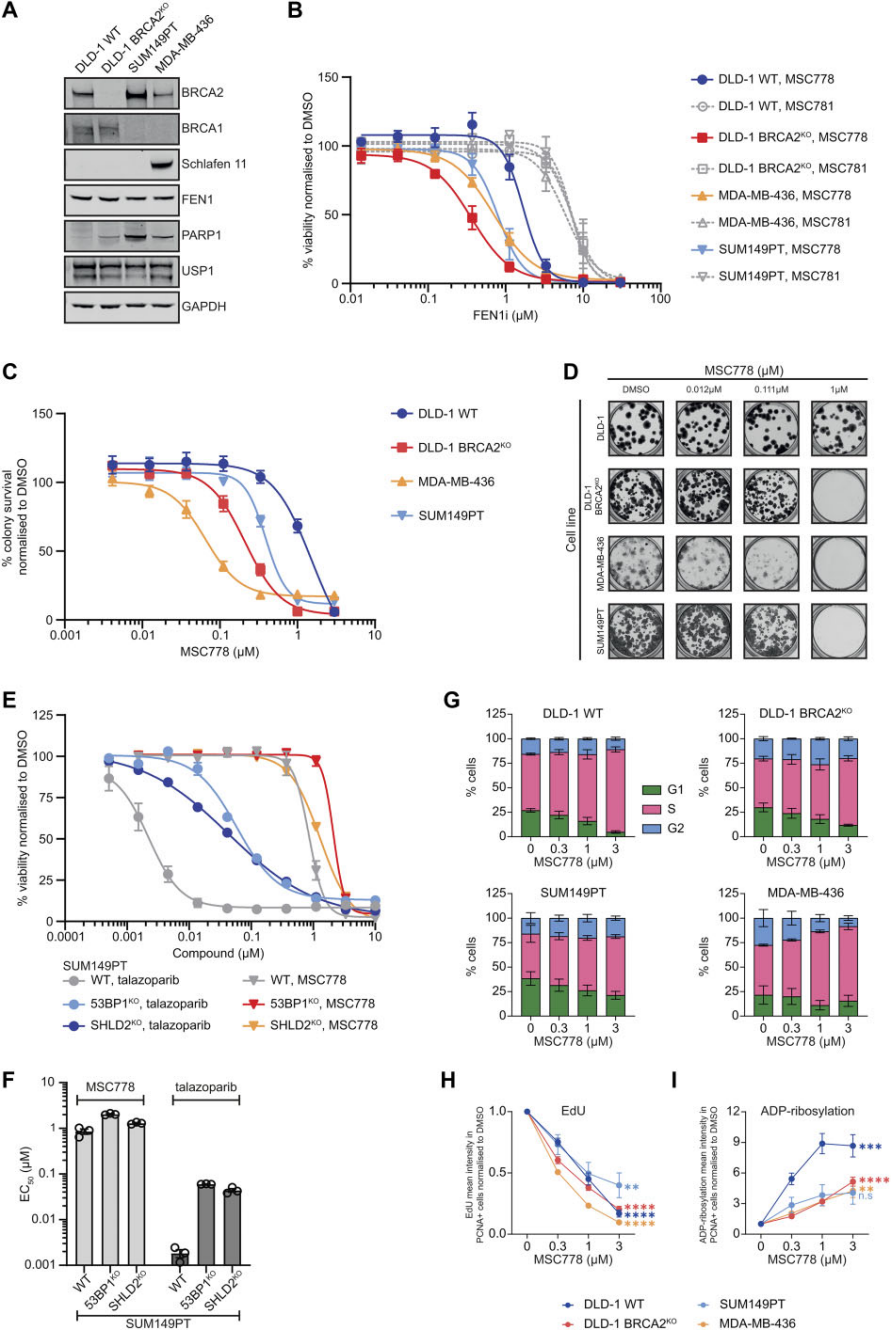
MSC778 selectively kills BRCA mutant cells and enriches cells in S-phase, suppresses active replication and increases S-phase ADP-ribosylation (**A**). Western blot analysis of BRCA mutant cell line panel: DLD-1, DLD-1 BRCA2^KO^, SUM149PT (BRCA1 Δ11q), and MDA-MB-436 (BRCA1 null). (**B**) Cell viability assays (CTG) were performed in a panel of BRCA mutant cell lines after 7 days treatment with indicated compounds. Data represent mean ± SEM of three biological replicates, each performed in technical triplicates. Summary data reporting EC_50_ values are shown in Supplementary Fig. S3A. (**C**) Colony survival assays were performed in a panel of BRCA mutant cell lines after treatment with FEN1 inhibitor MSC778. Data represent mean ± SEM of four biological replicates, each performed in technical triplicates. Summary data reporting EC_50_ values are shown in Supplementary Fig. S3B. (**D**) Representative images of colony survival assays upon treatment with MSC778. (**E**) Cell viability assays (CTG) were performed in the PARP inhibitor resistant SUM149PT 53BP1^KO^ and SHLD2^KO^ cell lines and their isogenic parental control (SUM149PT WT) after 7 days treatment with MSC778 or PARP inhibitor talazoparib. Data represent mean ± SEM of three biological replicates each performed in technical quadruplicates. (**F**) Summary data for MSC778 or PARP inhibitor activity in the cell panel used in panel (E). Bar plot represents the compound EC_50_ expressed as mean ± SEM of three biological replicates each performed in technical quadruplicates. (**G**) Cell cycle distribution after 24 h treatment with MSC778 was assessed in DLD-1, DLD-1 BRCA2^KO^, SUM149PT, and MDA-MB-436 cells. Cell cycle phase segments represent mean ± SEM of three biological replicates, each performed in technical triplicates. For each replicate sample *n* > 5000 cells were scored per condition. (**H**) Active replication, as marked by reduced EdU incorporation in S-phase cells and defined by PCNA staining, was measured after 24 h treatment with MSC778. Points represent mean ± SEM of three biological replicates, each performed in technical triplicates. For each replicate sample *n* > 1800 cells were scored per condition. Significance was determined using a one-way ANOVA test. (**I**) ADP-ribosylation in S-phase cells (defined by PCNA staining) was measured after 24 h treatment with MSC778. Points represent mean ± SEM of three biological replicates, each performed in technical triplicates. For each replicate sample *n* > 1800 cells were scored per condition. Significance was determined using a one-way ANOVA test.

Having observed killing of engineered *BRCA2*-deficient cells, we next investigated the sensitivity of cancer cell lines with mutations in *BRCA1*. SUM149PT and MDA-MB-436 are breast cancer cells harboring a hypomorphic Δ11q mutation or null mutation, respectively. *BRCA1/2* status was validated by western blot (Fig. 3A), and cell viability upon FEN1 inhibition was monitored using a 7-day viability assay. *BRCA1* mutation, like *BRCA2* loss conferred sensitization to MSC778 compared to DLD1 WT cells which served as an HR-proficient (HRP) control (Fig. 3B and Supplementary Fig. S3A). To account for the potential impact of doubling time on viability effects, we also performed colony survival assays in the same cells. As expected, *BRCA1/2* mutation conferred sensitivity to FEN1 inhibition, but we also observed a striking 10-fold enhancement in sensitivity of MDA-MB-436 cells (EC_50_ 65 nM), with more modest potency increases in DLD-1 BRCA2^KO^ and SUM149PT cells using this assay format (Fig. 3C and D, and Supplementary Fig. S3B).

To further investigate the breadth of MSC778 sensitivity in breast cancer cells, we performed viability screening in a dedicated cell panel (Supplementary Fig. S3C). Interestingly we observed significant trends in triple-negative breast cancer (TNBC) subtypes (Supplementary Fig. S3D and E) as well as HRD status (Supplementary Fig. S3F), the latter confirming our initial observations (Fig. 3B–D).

To further understand the potential application of FEN1 inhibition, we assessed MSC778 sensitivity in SUM149PT cells in which a targeted deletion of either *53BP1* or *SHLD2* confers resistance to the PARP inhibitor, talazoparib [50]. As expected, SUM149PT *53BP1*^KO^ and *SHLD2*^KO^ cells were ∼20-fold less sensitive than parental cells to talazoparib (Fig. 3E and F). However, we observed only a 2-fold impact on MSC778 sensitivity suggesting that mechanisms of PARP inhibitor resistance (such as mutations in the 53BP1–Shieldin axis [51–53]) may not substantially impact sensitivity to FEN1 inhibition and indeed PARP inhibitor resistant HRD cells could be targeted by FEN1 inhibitors.

**Figure 4.**
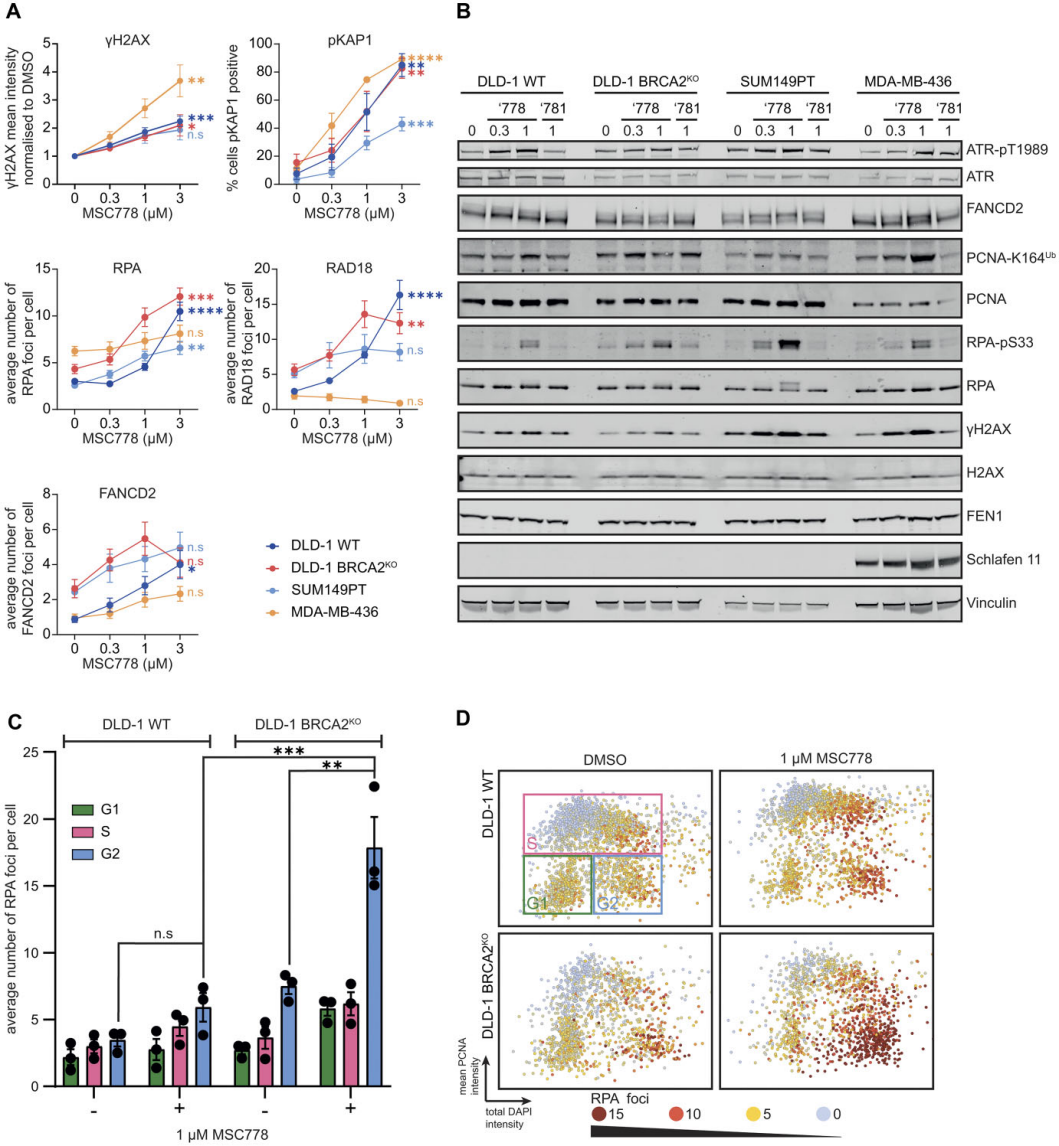
MSC778 induces DNA damage and elicits ssDNA gaps in G2 (**A**). Immunofluorescence of DDR markers γH2AX, pKAP1, RPA, RAD18, and FANCD2 in BRCA mutant cell line panel after treatment with indicated doses of MSC778 for 24 h. Points represent mean ± SEM of three biological replicates, each performed in technical triplicates. For each replicate sample *n* > 1400 cells were scored per condition. (**B**) Western Blot analysis of DDR markers after treatment with indicated doses of MSC778 or MSC781 for 24 h. Representative blot shown from two biological replicates. (**C**) RPA foci, indicating (post-replicative) ssDNA gaps, were measured in each cell cycle phase in DLD-1 and DLD-1 BRCA2^KO^ cells. Bars represent the mean ± SEM of three biological replicates, each performed in technical triplicates. For each replicate sample *n* > 650 cells were scored per condition. Significance was determined by a one-way ANOVA test followed by Tukey’s multiple comparison test. (**D**) Representative three-variable cell cycle plots of DLD-1 and DLD-1 BRCA2^KO^ cells upon treatment with 1 µM MSC778, indicating the enrichment of RPA foci in late S and G2 cells. Cell cycle distribution was determined by plotting total DAPI intensity against mean PCNA intensity. Points are coloured by number of RPA foci per cell.

To provide evidence of on-target mechanistic inhibition, we monitored cell cycle perturbations upon exposure to MSC778. In all four cell lines, including DLD-1 WT cells, a titratable S-phase accumulation (defined by PCNA staining) was observed, consistent with the essential role of FEN1 in DNA replication (Fig. 3G). DLD-1 *BRCA2*^KO^ and SUM149PT cells also showed an additional mild G2 accumulation, but MDA-MB-436 cells showed a dominant S-phase accumulation. These effects were not observed with MSC781 (Supplementary Fig. S4A). Dissecting this further, S-phase cells showed a substantial reduction in active DNA synthesis. Impaired progression through S-phase was marked by reduced EdU incorporation upon treatment with MSC778 (Fig. 3H and Supplementary Fig. S4B) and an elevation in ADP ribosylation (Fig. 3I and Supplementary Fig. S4B) indicating the activation of PARP-mediated SSBR signalling in response to replication inhibition by MSC778, albeit strongest in DLD-1 WT cells. Indirect immunofluorescence imaging demonstrated that MSC778 also elicited biomarkers of DNA damage. γH2AX and KAP1 pS824 (pKAP1 [54]) were induced in all four cell lines, but highest in MDA-MB-436. ssDNA exposure (RPA foci), translesion synthesis (RAD18 foci), and Fanconi Anaemia pathway activation (FANCD2) were all induced in DLD-1 WT, DLD-1 *BRCA2*^KO^, and SUM149PT cells (Fig. 4A and Supplementary Fig. S4B), indicative of replication perturbation and invocation of replication fork recovery mechanisms. However, induction of the latter markers in MDA-MB-436 was restricted, implying a modified cellular response to replication fork recovery in this cell line upon FEN1 inhibition. Western blot analysis (Fig. 4B) confirmed the induction of DNA damage and also activation of the S-phase checkpoint (ATR pT1989, pATR [55, 56]), RS (pRPA), and activation of RS-responsive monoubiquitination of PCNA and FANCD2, initiated to trigger DNA damage tolerance (DDT) pathways to bypass and/or fill-in ssDNA gaps post-replicatively. Supporting this latter hypothesis, we monitored the presence of RPA foci in different cell cycle phases upon MSC778 treatment, and observed increased foci in late S/G2 cells, which were substantially elevated in DLD-1 *BRCA2*^KO^ cells compared to DLD-1 WT controls (Fig. 4C and D, and Supplementary Fig. S4C). RPA foci induction in late S/G2 was also observed in SUM149PT cells but not in MDA-MB-436 cells (Supplementary Fig. S4C–E). MSC778 treatment ultimately induced cell death by apoptosis (Supplementary Fig. S4F and G). These data suggest that ssDNA gaps are formed in S, persist into G2, and are enriched in a *BRCA-*dependent manner upon FEN1 inhibition, where fork stabilization mechanisms are additionally compromised.

### Ewing sarcoma cells are hypersensitive to MSC778

**Figure 5.**
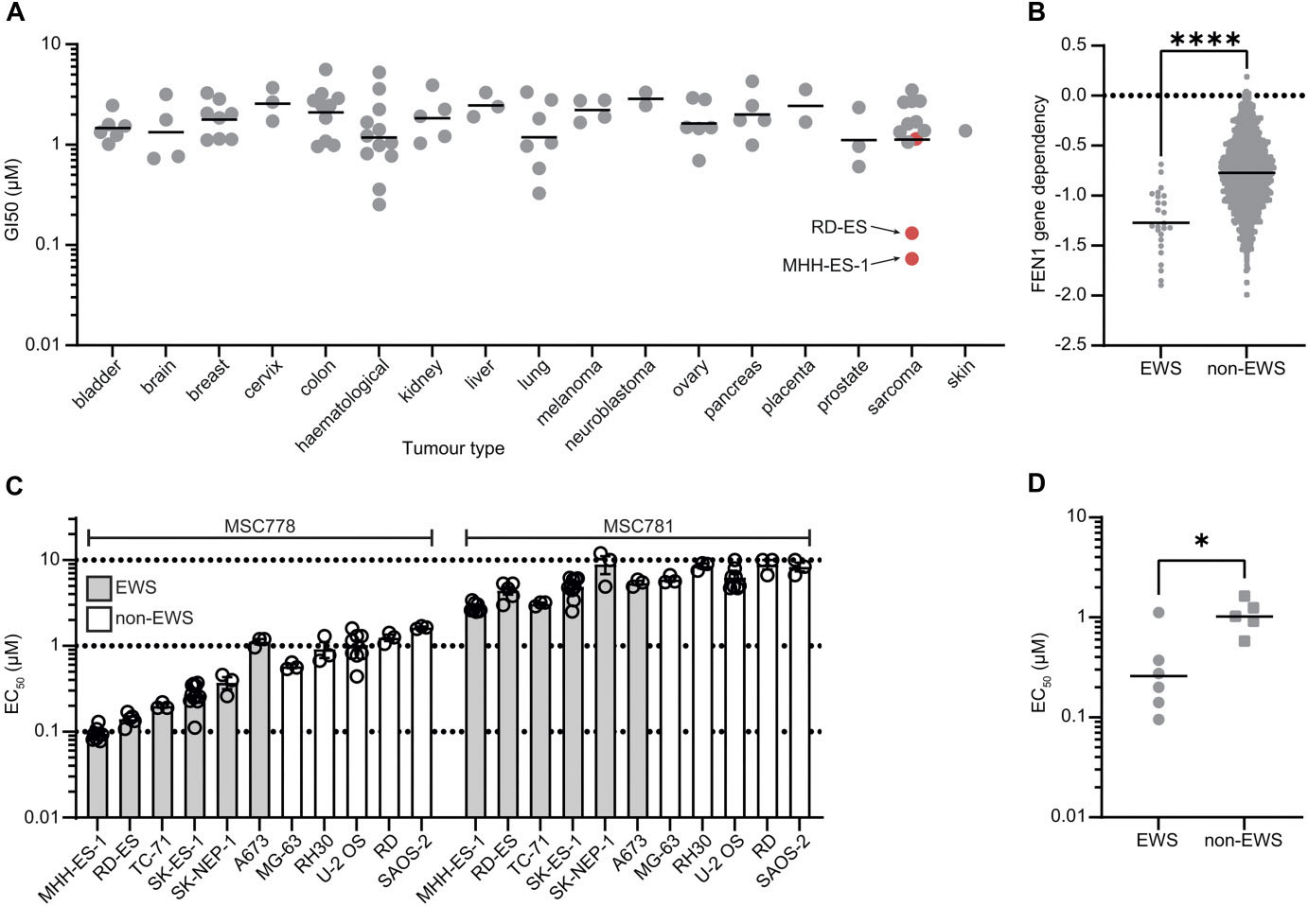
EWS cancer cells are hypersensitive to MSC778 (**A**). SRB-based cell growth/viability of 93 cancer cell lines in response to MSC778 was assessed at 5 days. GI_50_ values across the panel are represented by tumor type, with the geometric mean indicated. EWS cell lines are highlighted in red. Individual cell lines are shown in Supplementary Fig. S5A. (**B**) EWS cell lines have a significantly increased genetic dependency on FEN1 across all cell lines in DepMap. Significance was determined using a two-tailed *t*-test. (**C**) Cell viability assays were performed in a sarcoma cell line panel comprising EWS and non-EWS cell lines, after 7 days treatment with FEN1 inhibitor. Bar plot represents the compound EC_50_ expressed as mean ± SEM of ≥ 3 biological replicates each performed in technical triplicates. Data are combined from two panels (assayed by CTG or Alamar Blue) as outlined in Supplementary Fig. S5E. (**D**) Scatter plot showing EC_50_ values across the sarcoma panel with the geometric means indicated. Each data point represents mean ± SEM of ≥3 biological replicates each performed in technical triplicates. Significance was determined using a two-tailed *t*-test.

Having pharmacologically validated the BRCA synthetic lethality, we performed a cell panel screen covering a diverse set of tumor indications to identify potential cancer subtypes with sensitivity to MSC778 beyond BRCA1/2-mediated HRD. The two most sensitive cell lines were MHH-ES-1 and RD-ES (Fig. 5A and Supplementary Fig. S5A), both EWS models, prompting us to investigate EWS cells further.

Using publicly available datasets to assess gene dependency across cell lines, we observed that bone was the most sensitive tissue (Supplementary Fig. S5B), and within bone sarcoma lines, EWS models were highly dependent on FEN1 (Supplementary Fig. S5C). EWS models overall were the most sensitive tumor indication (Fig. 5B) and as previously reported also represent a population of high SLFN11 expression (Supplementary Fig. S5D), a notable biomarker of sensitization to replication associated DNA damage.

To validate these observations experimentally, we assessed the sensitivity of EWS and non-EWS sarcoma in viability assays. As anticipated, EWS cells were more sensitive to FEN1 inhibition than non-EWS cells (Fig. 5C and D, and Supplementary Fig. S5E), confirming EWS cells as an identifiable tumor indication with specific sensitivity to FEN1 inhibition.

### Biomarkers of response to MSC778 in EWS cells

**Figure 6.**
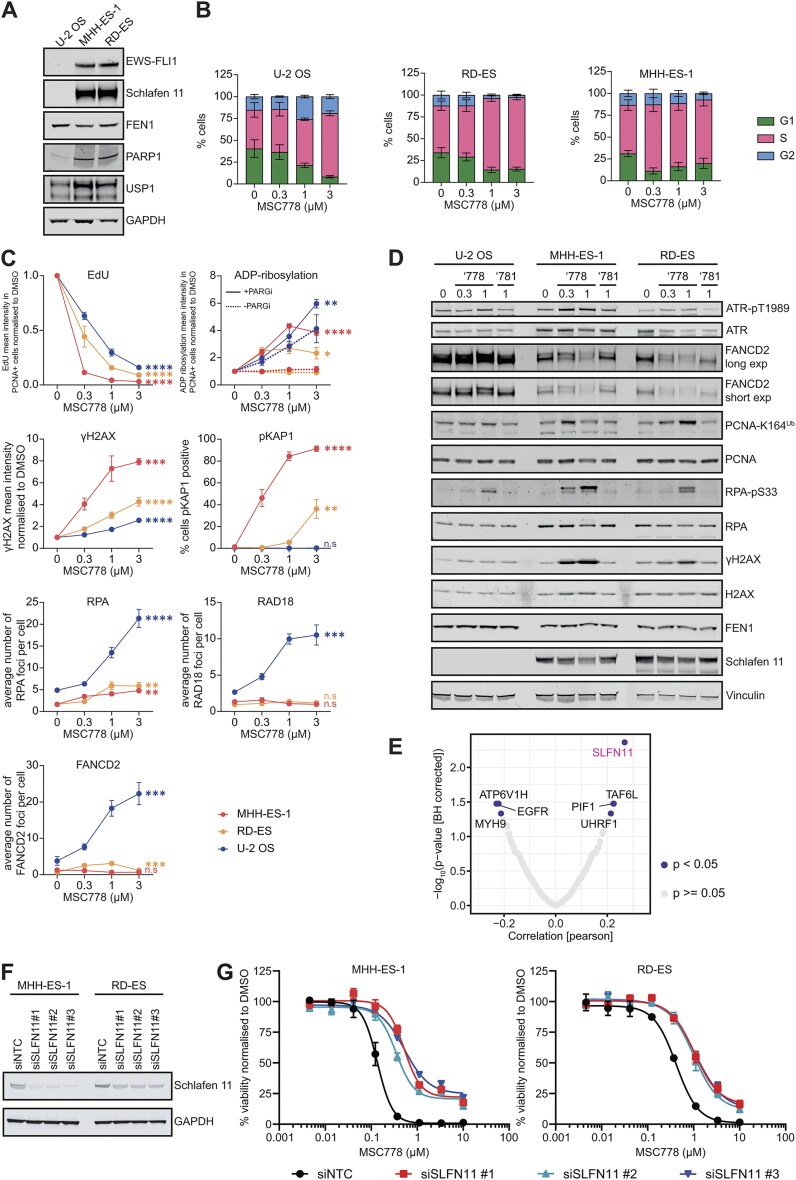
MSC778 enriches S-phase accumulation, suppresses active replication and increases DNA damage but does not elicit substantial ssDNA gap formation in EWS cell lines (**A**). Western blot analysis of EWS cell panel: U-2 OS (osteosarcoma, control), MHH-ES-1 (EWS), and RD-ES (EWS). (**B**) Cell cycle distribution after 24 h treatment with MSC778 was assessed in EWS and non-EWS cells. Cell cycle phase segments represent mean ± SEM of three biological replicates, each performed in technical triplicates. For each replicate sample *n* > 500 cells were scored per condition. (**C**) Immunofluorescence analysis of active replication (as marked by reduced EdU incorporation in S-phase cells and defined by PCNA staining), ADP-ribosylation in S-phase cells (defined by PCNA staining) ± PARG inhibition, and DDR markers (γH2AX, pKAP1 S824, RPA, RAD18, and FANCD2) in EWS and non-EWS cell lines after treatment with indicated doses of MSC778 for 24 h. Points represent mean ± SEM of three biological replicates, each performed in technical triplicates. For each replicate sample *n* > 250 cells were scored per condition. (**D**) Western Blot analysis of DDR markers after treatment with indicated doses of MSC778 or MSC781 for 24 h. Representative blot shown from two biological replicates. (**E**) SLFN11 expression positively correlates with sensitivity to MSC778 in a broad indication panel of 294 cell lines detailed in Supplementary Fig. S6C. (**F**) Western blot confirming siRNA-mediated knockdown of *SLFN11* in MHH-ES-1 and RD-ES cells. (**G**) Cell viability assays (CTG) were performed in MHH-ES-1 and RD-ES cells after 2 days treatment with indicated siRNAs and a further 4 days with MSC778. Data represent mean ± SEM of three biological replicates, each performed in technical triplicates.

To understand the mode of action of MSC778 in EWS cells, we characterized MHH-ES-1 and RD-ES cells upon exposure to MSC778 and compared them to U-2 OS cells, which served as a non-EWS, bone sarcoma control. Western blot analysis confirmed the expression of the *EWS–FLI1* fusion in EWS cells and the upregulation of some previously reported transcriptional targets Schlafen-11 [57], PARP1 [58–61], and USP1 [62, 63] (Fig. 6A). S-phase accumulation was observed in all three cell lines but was more pronounced in EWS cells (Fig. 6B and Supplementary Fig. S4A). U-2 OS cells showed mild G2/M accumulation, which was also earlier observed in non-SLFN11-expressing cell lines (Fig. 3G). MSC778 also induced apoptotic cell death in EWS cells (Supplementary Fig. S6A and B) but not in U-2 OS cells.

Although MSC778 impaired S-phase progression (decreased EdU incorporation), there was a lack of robust induction of ADP-ribosylation in EWS cells, which would be expected from the initiation of PARP-mediated SSBR upon OFM defects mediated by FEN1 inhibition (Fig. 6C). We reasoned that the lack of detectable PARylation may be due to the dynamic nature of PAR metabolism (balanced by opposing PARP1-mediated PARylation and PARG-mediated dePARylation) and indeed, inducible PARylation could be observed upon inhibition of PARG to promote PAR accumulation (Fig. 6C). Induction of γH2AX was weakest in U-2 OS, which also showed an absence of pKAP1 induction, likely through reduced DNA damage from elevated PARP-mediated SSBR (Fig. 6C). Activation of pATR, pRPA and monoubiquitination of PCNA and FANCD2 was observed in all three lines by western blot (Fig. 6D), but reduced in U-2 OS where functional repair likely ameliorates DNA damage accumulation.

Limited RPA, RAD18 and FANCD2 foci induction in EWS cells also suggested that replication inhibition was not inducing ssDNA exposure or DDT mechanisms (Fig. 6C). These observations are in agreement with previous studies suggesting that in *SLFN11*-expressing cells, Schlafen-11 is the dominant response factor to replication damage, triggering an irreversible replication block without extensive ssDNA exposure and RPA loading [64, 65]. The elevated PCNA monoubiquitination, without RAD18 foci, may indicate a misregulated TLS induction associated in response to RS. Of note, expression of *SLFN11* in MDA-MB-436 cells (Fig. 3A) may underly the similar trends in biomarker induction (such as limited RPA foci induction [32]) observed in response to MSC778.

Additional evidence for *SLFN11* expression as a biomarker of sensitivity to MSC778 came from a larger cell panel screen of ∼300 cell lines (Supplementary Fig. S6C). Bioinformatic analysis of sensitizing biomarkers in this screen, identified *SLFN11* mRNA expression as the top hit (Fig. 6E). This observation is confirmed in publicly available datasets, showing that *SLFN11* expression correlates with FEN1 gene dependency (Supplementary Fig. S6D). To confirm the role of *SLFN11* expression as a biomarker of sensitivity to FEN1 inhibition, we also assessed the impact of genetic depletion or ablation of *SLFN11* on viability upon treatment with MSC778. siRNA-mediated knockdown of *SLFN11* in MHH-ES-1 and RD-ES cells conferred increased resistance to MSC778 (Fig. 6F and G).

### CRISPR–Cas9 screening identifies determinants of MSC778 sensitivity

**Figure 7.**
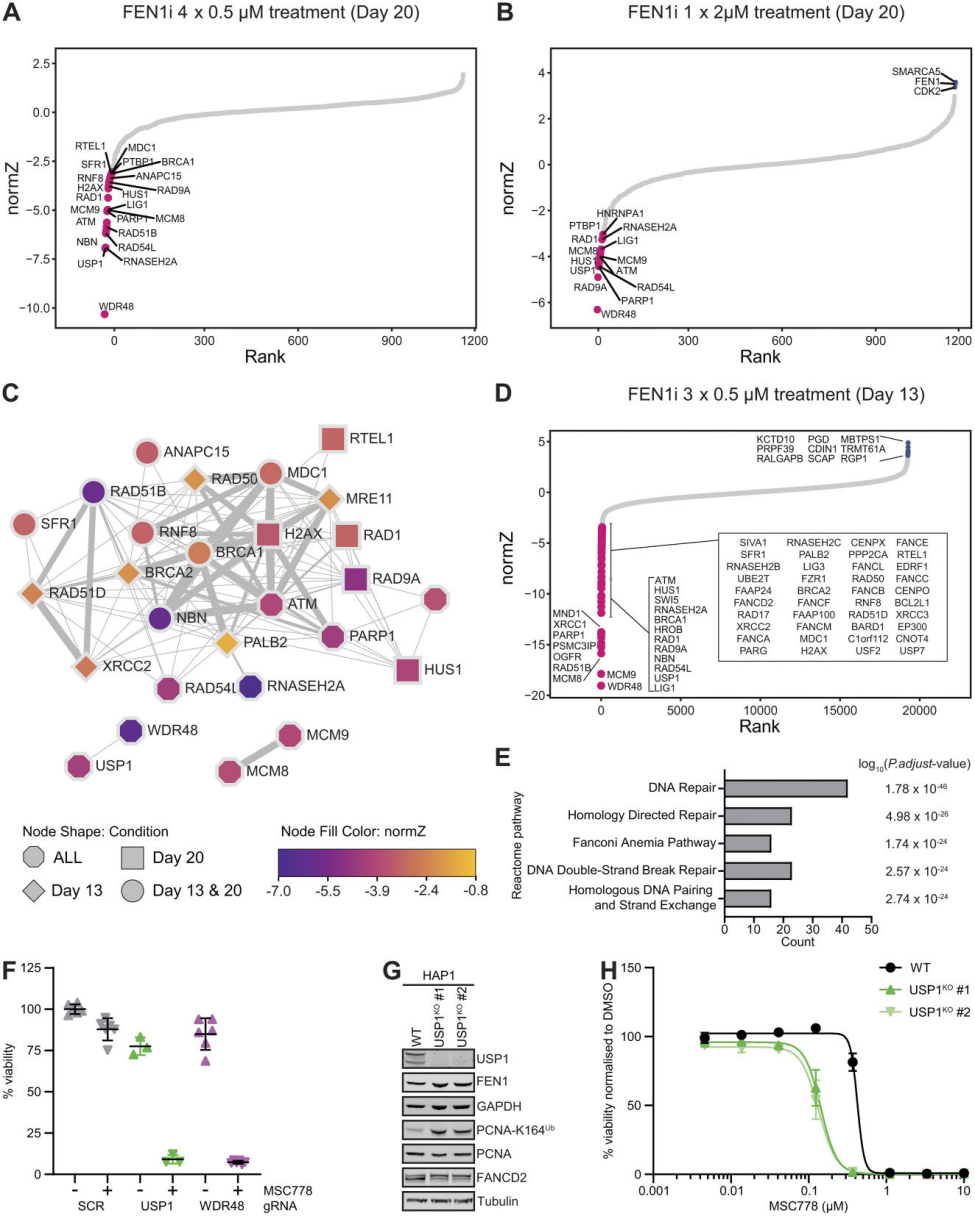
Inhibition of FEN1 creates a dependency on HR pathways and reveals a synthetic lethality with USP1–UAF1 (**A**). DrugZ analysis of sensitizers to MSC778 in RPE-1 cells identified using a DDR-targeted library. Genes enriched or depleted with FDR < 0.1 and a *Z-*score of >3 or < −3 in DrugZ analyses are highlighted in magenta and blue, respectively. Full guide counts are available in Supplementary Table S8. (**B**) DrugZ analysis of sensitizers and resistance factors to MSC778 in RPE-1 cells identified using a DDR-targeted library. Full guide counts are available in Supplementary Table S8. (**C**) Protein–protein interaction network map of consensus sensitizer hits scoring in three analysis methods using DDR-targeted CRISPR library. (**D**) DrugZ analysis of sensitizers to MSC778 in RPE-1 cells using a whole genome library. Full guide counts are available in Supplementary Table S9. (**E**) Reactome pathway enrichment analysis of sensitizer hits scoring across three analysis methods using a whole genome library. Top five pathways are shown. (**F**) Validation of synthetic lethality of USP1 and WDR48 with MSC778 using gRNAs. Data represent mean ± SD of technical replicates. (**G**) Western Blot analysis of HAP1^WT^ and two independent USP1^KO^ cell lines. (**H**) Cell viability assays (CTG) were performed in USP1^KO^ cells after 7 days treatment with MSC778. Data represent mean ± SEM of three biological replicates, each performed in technical triplicates.

To identify genetic determinants to pharmacological inhibition of FEN1, we used CRISPR–Cas9 chemosensitization screening in RPE1 *TP53*^KO^ cells. We initially used a focused library targeting ∼1000 DDR and related genes and multiple sampling conditions (Supplementary Fig. S7A) to capture both sensitizers and resistance factors coupled to analysis by three common methods (Supplementary Fig. S7B). Using a low dose exposure of MSC778, key factors in DSBR, specifically HR, were identified as sensitizers as anticipated (Fig. 7A, and Supplementary Figs S7C and S8A). We also observed that PARP1 loss sensitized to MSC778, mechanistically aligning with FEN1 inhibition triggering PARP-dependent SSBR. Unexpectedly, the top sensitizers included USP1 and WDR48 (Fig. 7A, and Supplementary Fig S8A and B), which function together as a critical deubiquitinase complex involved in DNA repair through deubiquitination of monoubiquitinated PCNA and FANCD2. Using a higher dose of MSC778, FEN1 was a clear resistance hit, providing high confidence in the specificity of MSC778 for its target (Fig. 7B, and Supplementary Figs S7C and S8C).

Building a consensus from these screens specifically identified key HR complexes such as BRCA1–BRCA2–PALB2 [16, 21], MRN [23], and the RAD51 paralogs, the 9-1-1 complex, MCM8/9 [66] as well as USP1–WDR48 [67, 68] (Fig. 7C) and these functional networks expanded when the consensus analysis criteria were relaxed (Supplementary Figs S9 and S10).

We next moved beyond the DDR target space, performing a genome wide CRISPR–Cas9 chemosensitization screen to identify additional sensitizers of the response to MSC778 (Supplementary Fig. S11A). Despite the increased genome coverage, sensitizers recovered from distinct analysis methods (Fig. 7D, and Supplementary Fig. S11B and C) remained strongly enriched in DNA repair factors, specifically HR (Fig. 7E), and aligned well to the DDR focused library screen results with additional support for FA pathway [23] and RNaseH2 components. Consistently, the USP1–WDR48 complex was a strong sensitizer to MSC778 (Fig. 7D). We further validated this observation using CRISPR–Cas9 mediated genetic ablation with gRNAs targeting USP1 and WDR48 to sensitize cells to MSC778 (Fig. 7F). Additionally, we used HAP1 cells to demonstrate a 3-fold sensitization of two independent USP1^KO^ cell lines to MSC778 compared to isogenic HAP1 WT cells (Fig. 7G and H).

Collectively, these data identify DDR factors, particularly the HR machinery, as the key pathways whose inactivation increases sensitivity to MSC778. Additionally, we provide strong evidence that the USP1–WDR48 complex constitutes an unexpected but robust synthetic lethal partner for FEN1 inhibition.

### MSC778 combines with multiple DDR inhibitors

We reasoned that the scope of DNA repair components identified through CRISPR–Cas9 screening, the mechanistic consequences of perturbing FEN1 functions in DNA transactions, and the mode of action of MSC778 (inhibiting FEN1 and enriching it on chromatin) would potentially be permissive to combination treatment with other DDR inhibitors (DDRi) and DNA damaging agents (DDAs).

**Figure 8.**
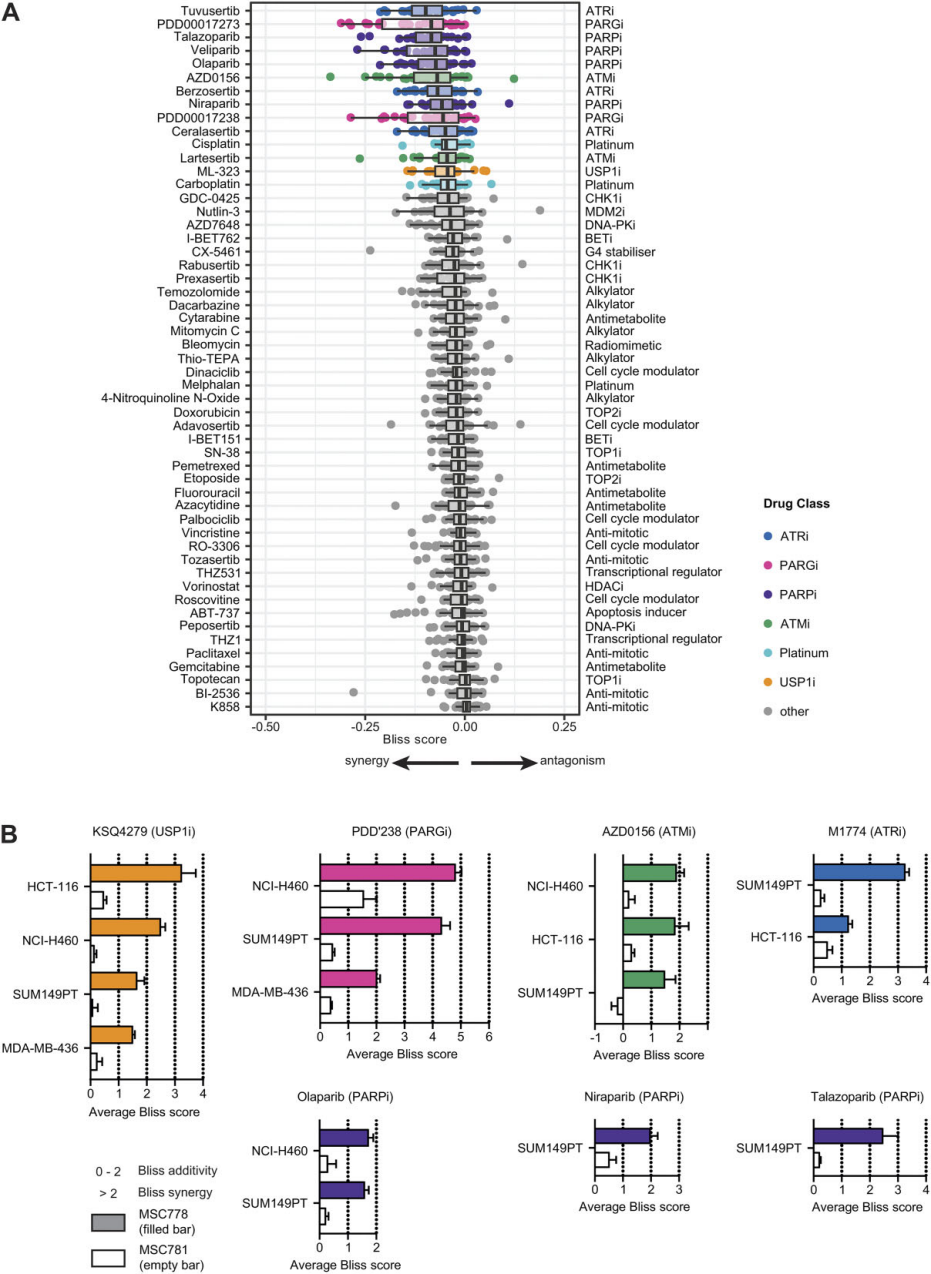
MSC778 combines with inhibitors of USP1, PARG, ATM, ATR, and PARP (**A**). Boxplots showing the treatment responses of drug combinations (MSC778 × combination partner) ranked by Bliss synergy responses in 34 cancer cell lines. Combination partner is on the left and its corresponding mode of action is on the right. The interquartile range (IQR) and median lines are shown, with whiskers extending to 1.5 × IQR. In this formulation the Bliss_excess_ is a continuous value between −1 and 1 and negative values are (potentially) synergistic, and positive values are (potentially) antagonistic. (**B**) MSC778 and MSC781 combinations with indicated combination partner compounds in indicated cell lines. Bars represent the mean Bliss synergy score ± SEM of *n* ≥ 3 biological replicates, each performed in technical duplicates. In this formulation the Bliss_excess_ is continuous and values higher than 2 are usually considered synergistic, and values between 0 and 2 are usually considered additive.

To test this hypothesis, and to validate the genetic interactions identified by CRISPR–Cas9 screening, a pharmacological screen was performed assessing viability of >30 cell lines upon exposure to a dose response of >50 DDRi/DDAs in the absence or presence of an anchor dose of MSC778. Across all cell lines, the most common drug classes identified as potential combination partners were inhibitors of ATR, PARG, PARP, ATM, platinum agents and notably ML323, an established tool compound targeting USP1 (Fig. 8A) [69, 70]. Although there was variability in the combination effects across cell lines, indicating that as yet unidentified biomarkers may drive context-specific combination efficacy with specific partners, the concordance with factors and mechanisms identified in this and our other screens was provocative.

We validated a range of combinations with MSC778 in cell lines selected from the screen for the robustness of their response. Combination benefits ranging from additivity to robust synergy were observed in both HRP (HCT-116, NCI-H460) and HRD (SUM149PT, MDA-MB-436) cells and were particularly strong with inhibitors of USP1 (KSQ-4279 [71]) and PARG (PDD00017238 [72]) (Fig. 8B, and Supplementary Fig. S12A and B). Similar combination effects on viability were not observed with MSC781. We also demonstrated combination efficacy using a range of PARP inhibitors in SUM149PT, which have reduced sensitivity to PARP inhibitor in monotherapy owing to the partial restoration of HR function conferred by the hypomorphic mutation in *BRCA1*, in contrast to the *BRCA*1 null status of MDA-MB-436 cells. As our initial validation was in HRD, focused on *BRCA1* mutation, we also assessed combinations in the isogenic pair of DLD-1 WT and DLD-1 *BRCA2*^KO^ cells to delineate if there was a differential response in *BRCA1* and *BRCA2* mutant cells. Inhibition of USP1 or PARG had strongly additive effects with MSC778 in DLD-1 *BRCA2*^KO^ cells, but ATR inhibition (Tuvusertib [73]) or a range of PARP inhibitors were at best weakly additive and no benefit was observed with ATM inhibition (AZD0156 [74]) (Supplementary Fig. S12C). Synergistic combination with USP1i and PARGi (PDD00017273 more so than PDD00017238) was, however, observed in DLD-1 WT cells (Supplementary Fig. S12C and D), highlighting the potential to combine FEN1 inhibition in HRP contexts. Inhibitors of ATR, ATM, and PARP had modest additivity in DLD-1 WT cells.

Building on our observation that MSC778 maintained monotherapy activity in engineered models of PARP resistance (Fig. 3E and F), we assessed whether combined inhibition of FEN1 and PARP was efficacious in this cellular context. MSC778 synergized with both olaparib (Supplementary Fig. S13A) and talazoparib (Supplementary Fig. S13B and C) in SUM149PT cells in which 53BP1 or SHLD2 were genetically ablated. Furthermore, MSC778 treatment reduced the concentration of PARP inhibitor required to suppress viability.

Given the hypersensitivity of EWS to MSC778 in monotherapy exposure, we also assessed the potential for combinations in the RD-ES cell line. Although limited to additivity, combination benefits were observed with PARP inhibitors, USP1 inhibitor, ATR inhibitor, and the PARG inhibitor PDD00017273 (Supplementary Fig. S13D). The observation of combination effects with both PARP and PARG inhibitors implies that pharmacological modulation of PAR metabolism is toxic in EWS cells. Additionally, as EWS cells have elevated levels of PARP1 (Fig. 6A), PARP inhibitors with a PARP “trapping” mode of action may have additional combination benefit through increased toxic replication fork stalling lesions. However, both “trapping” (olaparib, niraparib) and non-“trapping” (veliparib) PARP inhibitors show combination benefit with FEN1 inhibition, so the primary underlying mechanism is likely driven through enzymatic inhibition alone (Supplementary Fig. S13D and E).

## Discussion

**Figure 9.**
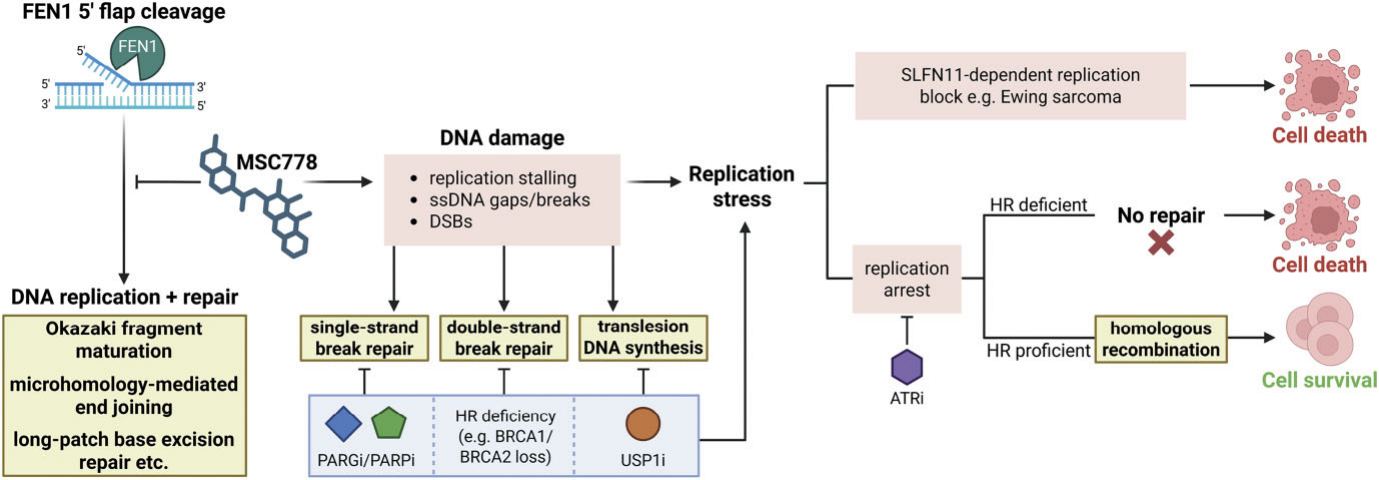
Model of MSC778 mechanism of action.

This study describes the characterization of a novel small molecule inhibitor of FEN1, MSC778, which invokes FEN1 chromatin recruitment (Fig. 2D, F, and G), suppresses DNA replication (Figs 3H and 6C), and selectively kills HRD and EWS cells (Figs 3B–D, Supplementary Fig. S3A and B, and Fig. 5C). In addition, MSC778 synergizes with inhibitors of PARP, PARG, USP1, and ATR (Fig. 8, and Supplementary Figs S12 and S13). These observations can be rationalized by the inhibition of known FEN1 functions in a variety of contexts as depicted in the model (Fig. 9).

The sensitivity of mutant BRCA cells to MSC778 either as monotherapy or in combination with PARP inhibitors can be explained in a number of ways. First, FEN1 activity contributes to efficient BER [15] and MMEJ [16] and suppression of either of these pathways is an established strategy to selectively kill HRD cells [75–77]. Second, chromatin enrichment of FEN1 may generate fork stalling lesions (such as those induced by PARP inhibition [78, 79]) which may contribute to the sensitization of *BRCA* mutant cells [12]. Third, unligated OFs, elicit RS and ssDNA gaps that are particularly toxic to *BRCA* mutant cells [80]. ssDNA gaps can also be processed into more toxic DSB lesions, or if allowed to accumulate, can exhaust cellular pools of RPA, which in turn leads to the persistence of under-replicated DNA into mitosis, and mitotic abnormalities that result in cell death. In the context of PARP inhibition, persistent ssDNA gaps in HRD cells may be due to the cells’ inability to repair PARP inhibitor-induced transcription–replication collisions [81]. Previous work implicated FEN1 activity in preventing such collisions [82] which may underlie the sensitization of BRCA mutant cells to PARP inhibitors when treated with MSC778. In addition, it has been shown that PARP inhibition reduces the integrity of nascent DNA strands which is exacerbated in FEN1-deleted cells [83]. The resulting post-replicative nicks or gaps may contribute to the increased sensitivity of BRCA null cells to PARP inhibition when co-administered with a FEN1 inhibitor.

Our studies aiming to expand the potential clinical reach of FEN1 inhibitors beyond the HRD setting, identified EWS cells and more generally, *SLFN11* expressing cancer cells, as potentially sensitive populations (Fig. 6E–G), which is consistent with the role of Schlafen-11 in response to RS-inducing DDRi/DDAs [84–86]. In *SLFN11* null cells, RS triggers an uncoupling of the replicative polymerase and helicase leading to extensive ssDNA exposure and RPA loading which recruits ATR-ATRIP and initiates a transient ATR-Chk1 mediated S-phase checkpoint, HR-mediated fork stabilization and repair [87, 88]. The absence of HR leads to extensive and persistent ssDNA gap formation in S/G2 and RPA exhaustion [89]. The presence of Schlafen-11 alters the response to RS [90, 91] in that RPA-bound ssDNA at stressed forks is directly bound by Schlafen-11 and triggers multiple events that irreversibly block replication including RPA filament disassembly [32, 90]. This is detectable as reduced RPA foci induction in EWS and MDA-MB-436 cells and the irreversible replication block results in cell death (Figs 4A and 6C, and Supplementary Fig. S4C and D). Mechanistically, whereas *SLFN11* expression appears to cause a pronounced S-phase accumulation in response to MSC778 (terminal replication blockage), in *SLFN11*-null cells the co-occurrence of a modest G2/M accumulation is also observed suggesting that partial exit from S-phase, albeit with damage, is feasible (Figs 3G and 6B) [92].

Data presented in this manuscript also identify additional settings where FEN1 inhibition may have monotherapy utility, beyond HRD or EWS/*SLFN11* expression. First, MDA-MB-436 cells, which carry a *BRCA1* null mutation, also express *SLFN11* (Fig. 3A), and this may contribute to the sensitivity of this cell line to FEN1 inhibition. We speculate that the co-occurrence of *BRCA* mutation and *SLFN11* expression may represent a population with enhanced sensitivity to FEN1 inhibition [93–95]. Second, *53BP1* or *SHLD2* deletion in the context of *BRCA1* mutation causes PARP inhibitor resistance but does not robustly affect the efficacy of MSC778 (Fig. 3E and F). This suggests that some PARP inhibitor resistant tumors may be targetable by FEN1 inhibitors. We also demonstrate a synergistic *in vitro* efficacy between FEN1 and PARP inhibitors in these engineered PARP resistant models (Supplementary Fig. S13A–C) suggesting that this combination may overcome a subset of PARP resistance mechanisms. It will be important to investigate whether combining both FEN1 and PARP1 inhibitors can overcome other forms of PARP resistance.

In addition to expanding the potential for FEN1 inhibitors as a monotherapy, our CRISPR–Cas9 chemosensitization (Fig. 7A–F and Supplementary Figs S7–11) and pharmacological combination screens (Fig. 8, and Supplementary Figs S12 and S13) demonstrated synergy between MSC778 and DDRi targeting PARP, PARG, ATR, ATM, and USP1.

Mechanistically, these synergies are likely driven by three consequences of FEN1 inhibition: the persistence of SSBs and ssDNA gaps when 5′-flap cleavage is prevented, the disruption to DNA repair mechanisms engendering a reliance on back-up pathways, and the suppression of OFM triggering RS.

OFM defects are a major source of ssDNA lesions and are tightly linked to PAR metabolism which is in turn highly regulated by opposing activities of PARP1 and PARG [7, 96, 97]. Thus, the combination with PARP and PARG inhibitors can be rationalized by the role of FEN1 in OFM. Support for combination of FEN1 inhibition with PARG inhibition is provided by several recent reports highlighting genetic synthetic lethality between PARG and BER/OFM factors as well as chemosensitization screens showing that FEN1 loss strongly sensitizes to PARG inhibitors [96]. Three PARG inhibitors are currently in clinical trials (NCT06395519, NCT05787587, and NCT06614751) focusing on advanced solid tumors with HRD and/or DDR deficiencies. Combination with FEN1 inhibition could broaden the therapeutic potential of PARG inhibitors in both HRD and HRP contexts.

Various ATR inhibitors are currently under clinical evaluation in both monotherapy and combination settings [73, 98–102] and thus the potential for synergies with FEN1 inhibitors has clear translational significance. We have demonstrated that FEN1 inhibition triggers RS, which activates the ATR-dependent S-phase checkpoint and this likely forms the basis of the combination effect. It will be important to understand the impact of *SLFN11* expression, which interferes with, and can be dominant over, the ATR-mediated response.

USP1 inhibitors have recently emerged as potential anti-cancer agents and we validated the deubiquitinase activity of USP1–WDR48 as a robust synthetic lethal partner for MSC778 using both genetic and pharmacological approaches (Figs 7F–H and 8, and Supplementary Figs S12 and S13D, E). The normal function of USP1 is in the deubiquitination of PCNA [103] and FANCD2 [67, 68], which are monoubiquitinated by RAD18 and the FA core complex respectively, in response to RS. The induction of these modifications is essential for both TLS and FA pathway repair but their persistence upon USP1 inhibition leads to misregulated activation [104], leading to replication fork instability and excessive ssDNA gap formation [105]. This is particularly deleterious in a *BRCA* mutant context where fork integrity is already compromised [106]. USP1 inhibitors may also induce fork stalling lesions by preventing its autocleavage and trapping USP1 on DNA [105, 107] and these combined effects on replication dynamics likely create a dependency on FEN1 function in BER. We have demonstrated that MSC778 can activate TLS, but if the TLS repair response is subsequently misregulated by USP1 inhibition [105, 106], the combined inactivation of both FEN1 and USP1 would add an additional component to the observed synthetic lethality and aligns to recent data confirming this USP1–FEN1 genetic interaction [108].

Although USP1 inhibitor anchored chemosensitization screens have been performed in HRD cells (BRCA1 Δ11q UWB1.289 cells [71, 105]), our data provide strong evidence that combined inhibition of FEN1 and USP1 may work in HR-proficient settings. Combined USP1 and PARP1 inhibition has already demonstrated activity, including in a model of PARP resistance [71], and it will be informative to compare the combination efficacy with FEN1 inhibitors in these and other settings. Current clinical studies are evaluating USP1 inhibitors alone or in combination with olaparib or carboplatin (NCT05240898 [71]), and a FEN1 inhibitor could represent a promising novel combination partner.

The potential for ssDNA lesions to become DSBs may underpin the combination with ATM inhibitors. However, the observation of an S-phase accumulation and associated RS triggering ATR and/or Schlafen-11 responses may limit the contexts or strength of combination with FEN1 inhibition. Beyond EWS, the broader importance of *SLFN11* expression as a sensitivity biomarker in cancer should be investigated, to clarify the threshold of expression required to elicit sensitivity.

In summary, we have presented evidence of combination potential, ranging from additivity to strong synergy, with a variety of DDRi. *In vitro* efficacy has been observed independent of HR status, opening up the scope of both FEN1 inhibitors and putative combination partner DDRi beyond HRD cancers. It will be critical to understand how broadly these combinations can be applied and whether biomarkers can be identified to support their translation into precision oncology strategies.

We speculate that monotherapy activity is driven by inhibition of DNA replication and repair, providing a foundation for targeting HRD and DDR defective cancer cells with an altered RS response e.g. *SLFN11* high. In *SLFN11*-null settings, FEN1 inhibition also induces ssDNA gap formation, which is an emerging area of high interest and also a feature of PARP [109], PARG [97], and USP1 [105] inhibition. Combination potential may be more effective in the context of S-phase checkpoint inhibition (additionally impacted by *SLFN11* expression) or factors that themselves induce ssDNA gaps. This latter option may exacerbate ssDNA lesions to excessive levels that compromise the viability of cancer cells beyond HRD [10, 12].

Collectively, our data characterize a novel small molecule inhibitor of FEN1 and reveals the therapeutic potential of FEN1-targeting medicines.

## Supplementary Material

gkaf1279_Supplemental_Files

## Data Availability

All data needed to evaluate the conclusions of this study are presented in the article and/or the Supplementary Data. The data will be shared on reasonable request to the corresponding author.
