# Supplementary material for: Mechanistic insights into the monotherapy and combination potential of FEN1 inhibition in cancer therapy
Source: Nucleic Acids Res. 2025 Dec 8;53(22):gkaf1279. doi: 10.1093/nar/gkaf1279 (PMC12684391; doi:10.1093/nar/gkaf1279)
Supplement: gkaf1279_Supplemental_Files [file gkaf1279_supplemental_files.zip › Rajendra et al Supplementary Data - revision.pdf]

SUPPLEMENTARY DATA

Mechanistic insights into the monotherapy and combination potential of FEN1 inhibition in cancer therapy

SUPPLEMENTARY FIGURES

- Supplementary Figure 1. Recombinant proteins used for biochemical assays and the effect of MSC781 on viability of DLD-1 and DLD-1 BRCA2^{KO} cells
- Supplementary Figure 2. Characterisation of MSC778 modulation of FEN1 in chromatin retention and MMEJ reporter assays
- Supplementary Figure 3. Sensitivity to FEN1 inhibition correlates with homologous recombination deficiency in a breast cancer cell line panel
- Supplementary Figure 4. Cell cycle analysis control experiments using MSC781, representative images of DDR marker induction, RPA foci induction in *BRCA1* mutant cell lines, and induction of apoptosis
- Supplementary Figure 5. EWS cells are pharmacologically sensitive to inhibition of FEN1 and genetically dependent on FEN1
- Supplementary Figure 6. Cell panel screening identifies Ewing sarcoma and SLFN11 expression as biomarkers of response to MSC778
- Supplementary Figure 7. DDR-targeted CRISPR screens with MSC778 sampled with indicated doses at indicated timepoints using multiple analysis methods
- Supplementary Figure 8. Examples of genetic interactions identified using DDR-targeted CRISPR library screens with MSC778
- Supplementary Figure 9. Expanded consensus of sensitisers to MSC778 identified using DDR-targeted library CRISPR screens
- Supplementary Figure 10. Overlap of hits scoring in DDR-targeted library CRISPR screens
- Supplementary Figure 11. Whole genome CRISPR screen with MSC778
- Supplementary Figure 12. MSC778 synergises with inhibitors of USP1 and PARG in HRD and HRP cell lines
- Supplementary Figure 13. MSC778 synergises with PARP inhibitors in genetically engineered models of PARP resistance and RD-ES cells

SUPPLEMENTARY TABLES (METHODS)

- Supplementary Table 1: Substrate oligonucleotides
- Supplementary Table 2: Cell lines and culturing media
- Supplementary Table 3: Compounds
- Supplementary Table 4: Combination matrices
- Supplementary Table 5: Antibodies
- Supplementary Table 6: Guide RNAs
- Supplementary Table 7: Software

SUPPLEMENTARY TABLES (DATA)

- Supplementary Table 8: Focused library CRISPR screen gRNA counts
- Supplementary Table 9: Whole genome CRISPR screen gRNA counts

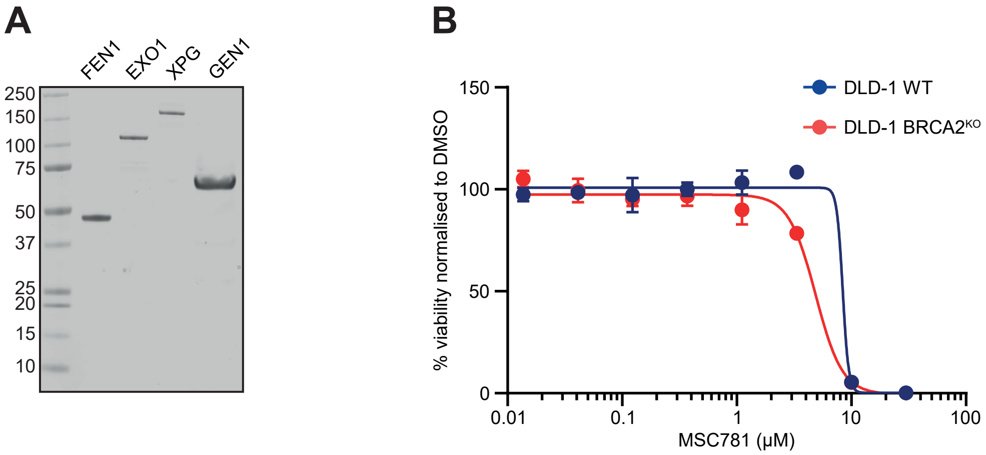

FEN1 - Supplementary Figure S1

Supplementary Figure 1 Recombinant proteins used for biochemical assays and the effect of MSC781 on viability of DLD-1 and DLD-1 BRCA2^{KO} cells

A. SDS-PAGE gel showing recombinant protein used in biochemical assays.
B. Cell viability assays (AlamarBlue) were performed in DLD-1 and DLD-1 BRCA2^{KO} cells after 7 days treatment with MSC781. Data represent mean ± SEM of 3 biological replicates, each performed in technical duplicates. Corresponding data with MSC778 are shown in Figure 1I.

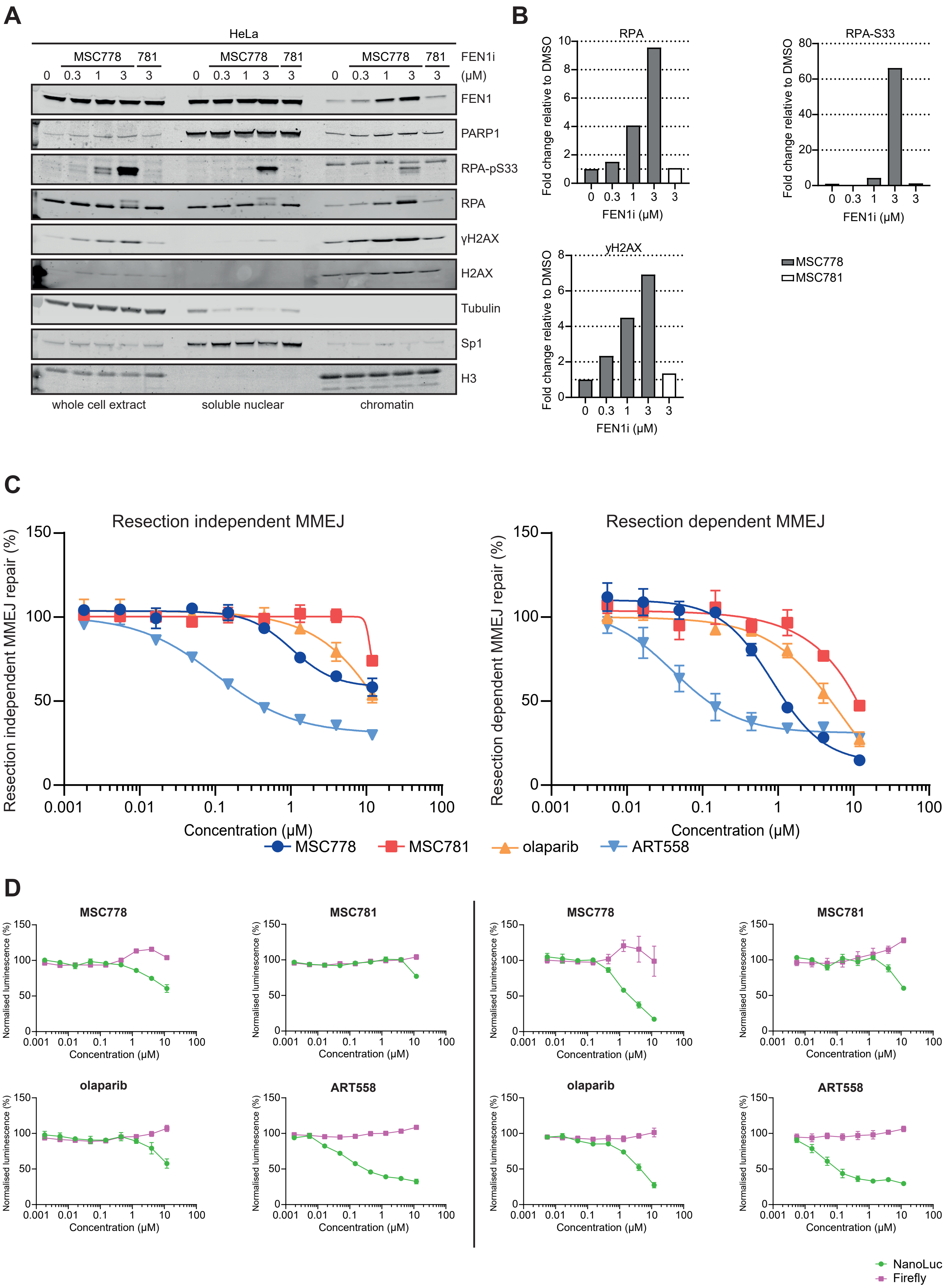

FEN1 - Supplementary Figure S2

Supplementary Figure 2. Characterisation of MSC778 modulation of FEN1 in chromatin retention and MMEJ reporter assays

A. Full Western blot analysis of subcellular fractionation of HeLa cells shown in Figure 2G.
B. Western blot quantification for data shown in Figure 2G. Data represent the signal intensity for the indicated proteins relative to H3 normalised to DMSO control.
C. MSC778 inhibitor suppresses MMEJ in extrachromosomal reporter assays. Extrachromosomal MMEJ reporter assays were performed in HEK-293 cells treated with increasing concentrations of MSC778, MSC781, PARP inhibitor, olaparib, and Polθi, ART558. The inhibition of MMEJ, in a dose-dependent manner, was detected using two distinct reporter substrates independent (left) or dependent (right) on resection prior to MMEJ.
D. Percentage inhibition of the individual luminescence signals, shown below, was calculated relative to the DMSO control.

Data represent mean ± SEM of 3 biological replicates each performed in technical quadruplicates, fitted with a non-linear regression curve (four parameters).

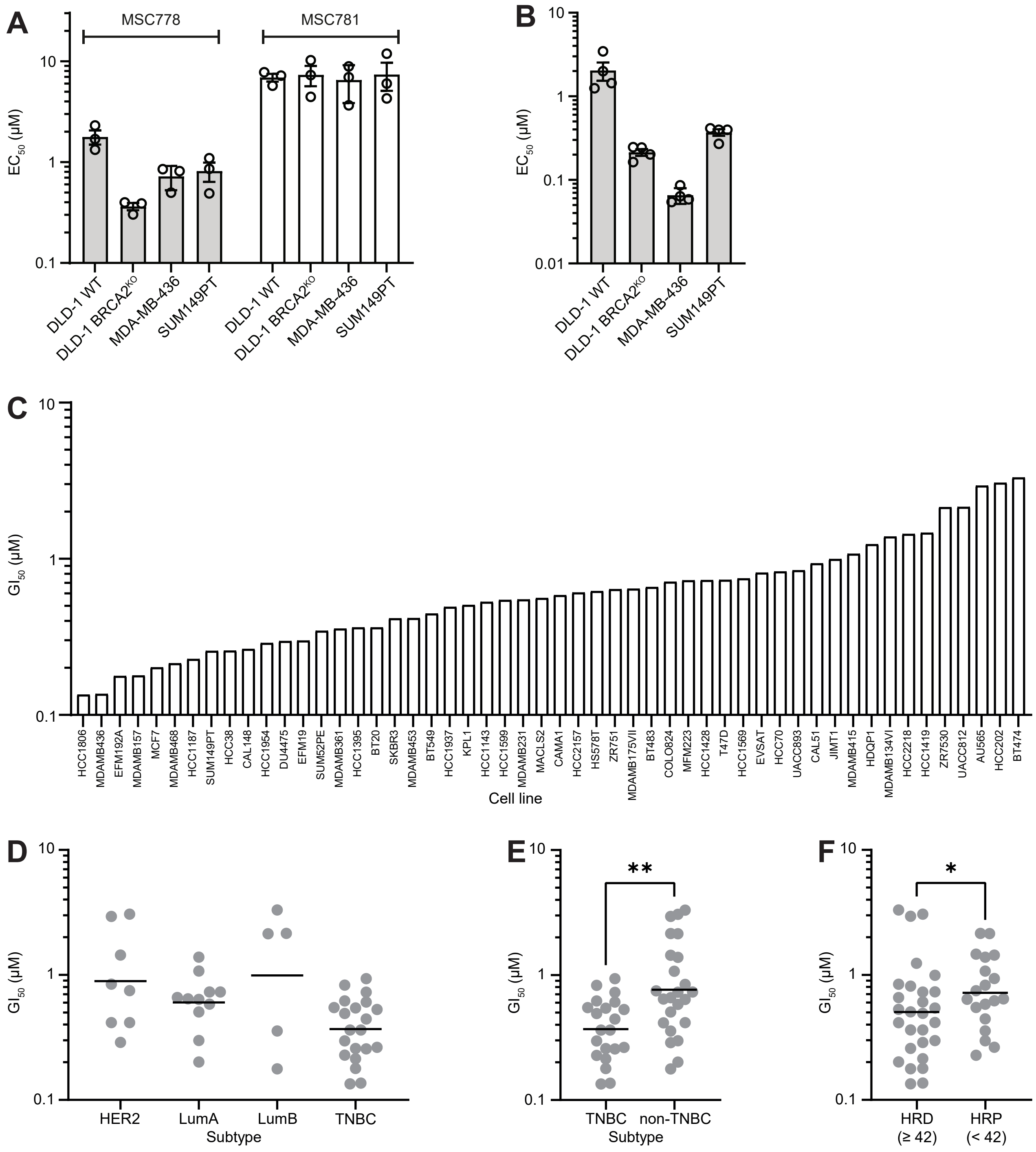

FEN1 - Supplementary Figure S3

Supplementary Figure 3. Sensitivity to FEN1 inhibition correlates with homologous recombination deficiency in a breast cancer cell line panel

A. Summary data reporting the EC_{50} for MSC778 and MSC781 in the BRCA mutant cell panel shown in Figure 3B. Bars represent the mean ± SEM of three biological replicates, each performed in technical triplicates.
B. Summary data reporting the EC_{50} for the MSC778 in the BRCA mutant cell panel shown in Figure 3C. Bars represent the mean ± SEM of 4 biological replicates, each performed in technical triplicates.
C. Assessment of FEN1 sensitivity across a breast cancer cell line panel. Sulforhodamine B (SRB)-based cell growth/viability of 52 breast cancer cell lines in response to FEN1 was assessed at 7 days. GI_{50} values across the panel are represented by individual cell line.
D. GI_{50} values for 45/52 cell lines collated by cancer subtype, with the geometric means indicated.
E. GI_{50} values for 45/52 cell lines collated by cancer subtype grouped as TNBC or non-TNBC, with the geometric means indicated. Significance was determined using an unpaired two-tailed t-test.
F. GI_{50} values for 47/52 cell lines collated by HRD score, with the geometric means indicated. Significance was determined using a one-tailed Mann-Whitney test.

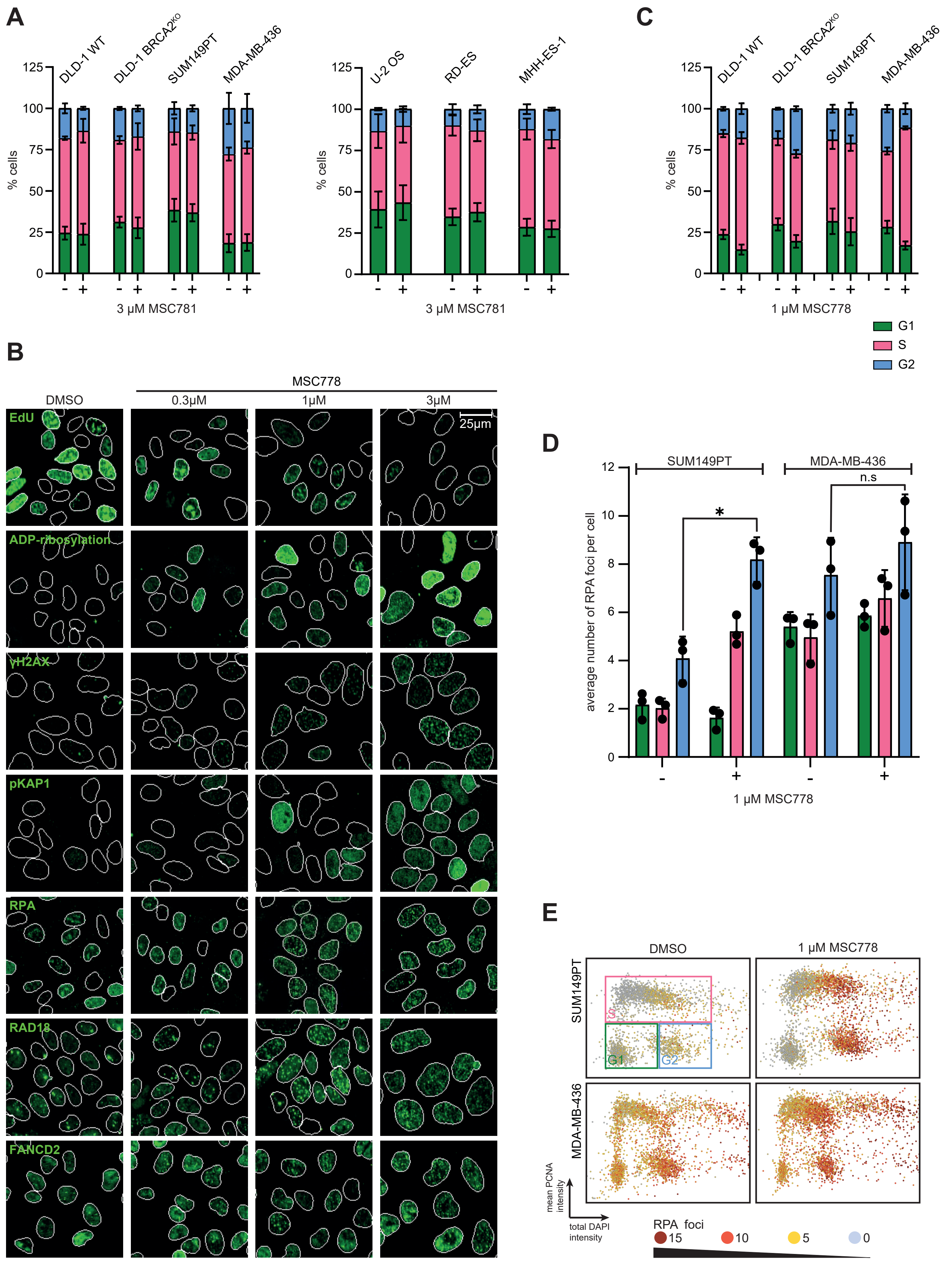

FEN1 - Supplementary Figure S4

F

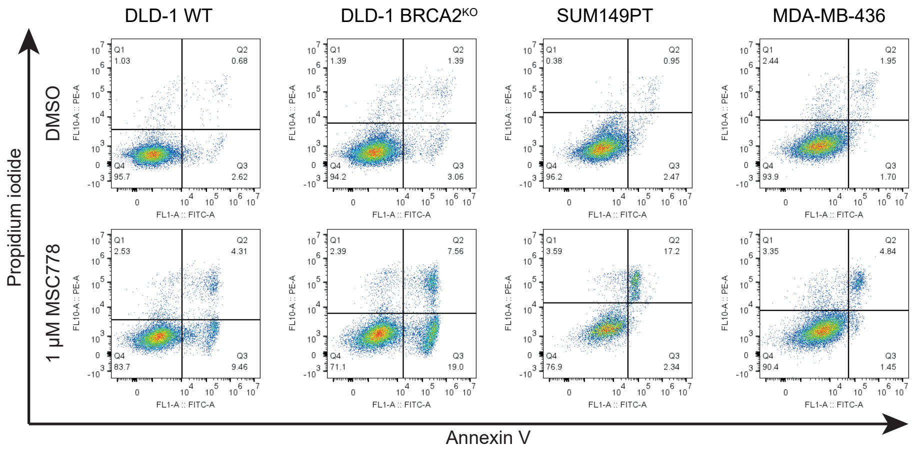

G

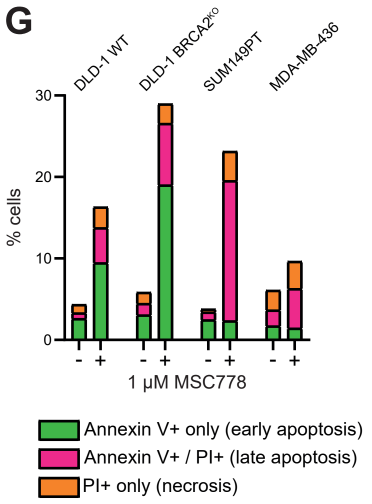

FEN1 - Supplementary Figure S4 continued

Supplementary Figure 4. Cell cycle analysis control experiments using MSC781, representative images of DDR marker induction, RPA foci induction in *BRCA1* mutant cell lines, and induction of apoptosis

A. Cell cycle of cell lines in HRD panel (DLD-1, DLD-1 BRCA2^{KO}, SUM149PT, MDA-MB-436) and EWS panel (U-2 OS, RD-ES and MHH-ES-1) are not perturbed by 3 μM MSC781. For each replicate sample n>4000 cells were scored per condition.
B. Representative images of the indicated DDR markers detected by immunofluorescence following MSC778 treatment in DLD-1 BRCA2$^{-/-}$ cells
C. Cell cycle analysis staining using anti-PCNA antibody AB18197 (co-stained with RPA) is comparable to analysis performed with anti-PCNA antibody PC10 (co-stained with ADP-ribose and EdU) in Figure 4C, 4D, S4C and S4D. For each replicate sample n>600 cells were scored per condition.

Cell cycle distribution segments represent mean ± SEM of three biological replicates, each performed in technical triplicates.

D. RPA foci, indicating (post-replicative) ssDNA gaps, was measured in each cell cycle phase in SUM149PT and MDA-MB-436 cells. Bars represent the mean ± SEM of three biological replicates, each performed in technical triplicates. For each replicate sample n>1000 cells were scored per condition.
E. Representative three-variable cell cycle plots of SUM149PT and MDA-MB-436 cells upon treatment with 1 μM MSC778, indicating the enrichment of RPA foci in late S and G2 cells in SUM149PT cells. Cell cycle distribution was determined by plotting total DAPI intensity against mean PCNA intensity. Points are coloured by number of RPA foci per cell.
F. Bivariate plots of Annexin V vs PI intensities assessed by flow cytometry. Cells were classified as healthy (Annexin V^{-}/PI^{-}), necrotic (PI^{+} only), early apoptotic (Annexin V^{+} only) and AnnexinV^{+}/PI^{+} (late apoptotic). Data are representative of 2 biological repeats.
G. Percentage of cells displaying markers of cell death (Annexin V^{+} only, PI^{+} only or Annexin V^{+}/PI^{+}). Data are representative of 2 biological repeats.

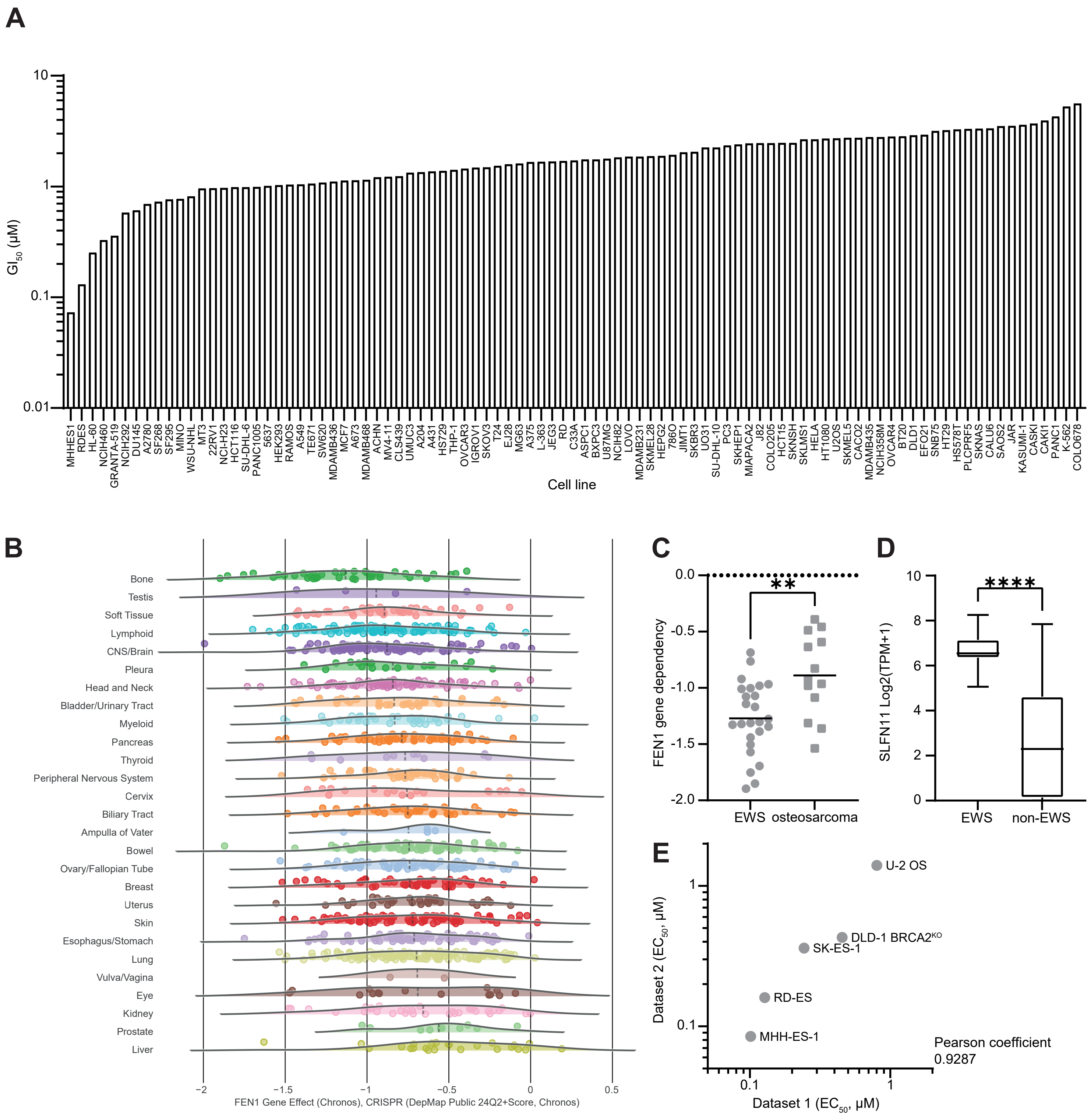

FEN1 - Supplementary Figure S5

Supplementary Figure 5. EWS cells are pharmacologically sensitive to inhibition of FEN1 and genetically dependent on FEN1

A. Sensitivity of individual cell lines in cell panel shown in Figure 5A. Viability was determined by SRB-based cell growth/viability in response to 5 day treatment with MSC778.
B. Genetic dependency on FEN1 across cell lines in DepMap, grouped by tissue type. Bone cells lines have the highest dependency. Tissue groups containing a single cell line have been excluded.
C. Among bone sarcoma cell lines, Ewing sarcoma cell lines have a significantly increased genetic dependency on FEN1. Significance was determined using a two-tailed t-test.
D. Ewing sarcoma cell lines have a significantly increased expression of SLFN11. Significance was determined using a two-tailed t-test.
E. Data from 2 independent cell panels (7 days, set 1 = CTG, set 2 = AlamarBlue) were merged to generate data in Figure 5C. EC_{50} values robustly correlated across both datasets.

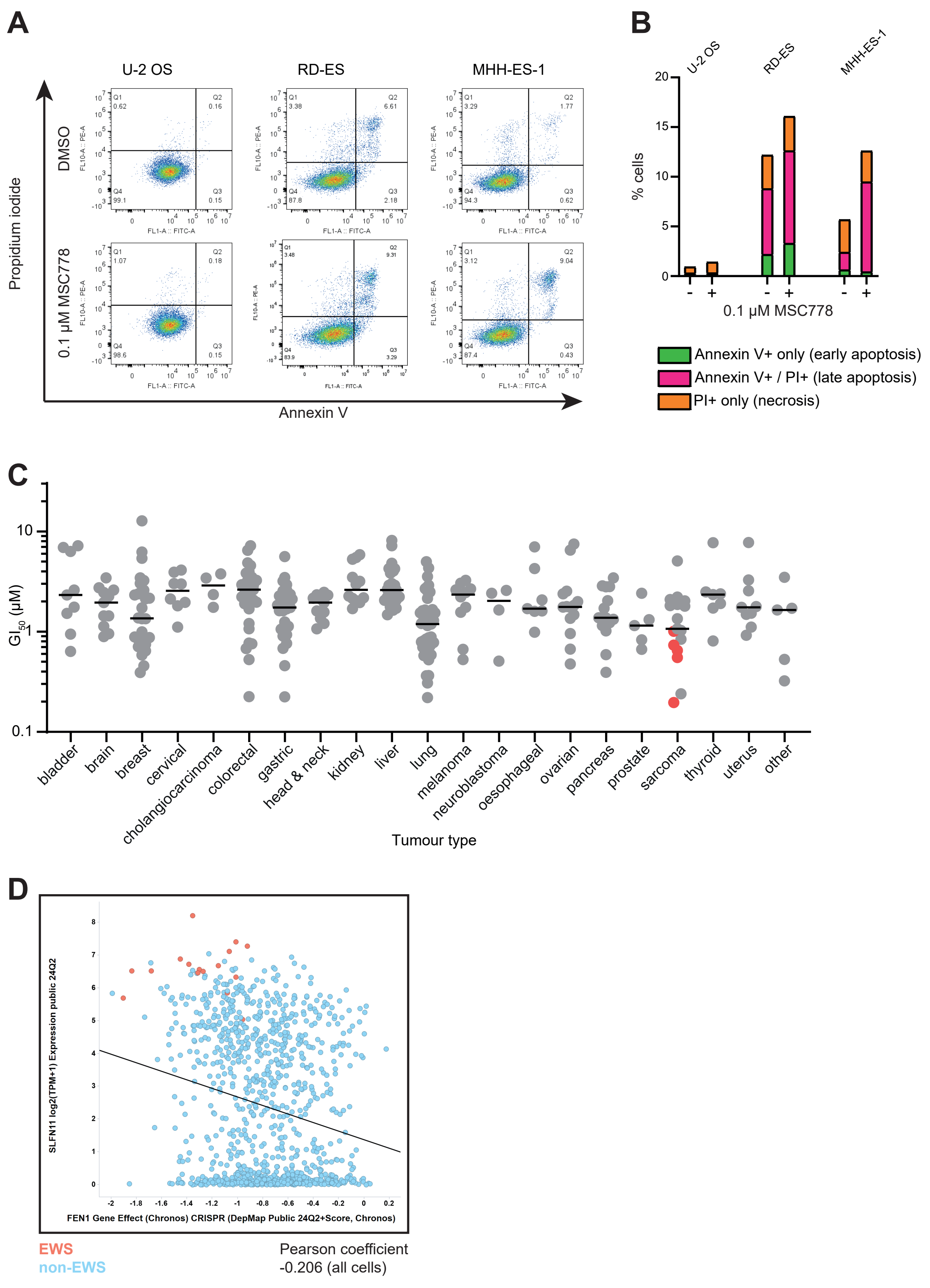

FEN1 - Supplementary Figure S6

Supplementary Figure 6. Cell panel screening identifies Ewing sarcoma and SLFN11 expression as biomarkers of response to MSC778

A. Bivariate plots of Annexin V vs PI intensities assessed by flow cytometry. Cells were classified as healthy (Annexin V^-/PI^-), necrotic (PI^+ only), early apoptotic (Annexin V^+ only) and Annexin V^+/PI^+ (late apoptotic). Data are representative of 2 biological repeats.
B. Percentage of cells displaying markers of cell death (Annexin V^+ only, PI^+ only or Annexin V^+/PI^+). Data are representative of 2 biological repeats.
C. Assessment of FEN1 sensitivity across a broad indication panel of 299 cell lines. CellTiter-Glo (CTG)-based cell growth/viability in response to FEN1 was assessed at 10 days. GI_{50} values across the panel are represented by tumour type, with the geometric mean indicated. Ewing sarcoma cell lines are highlighted in red. “Other” combines data from adrenal, lymphoma, retinoblastoma, small intestine cell lines.
D. Increased SLFN11 expression correlates with increased genetic dependency on FEN1 in DepMap.

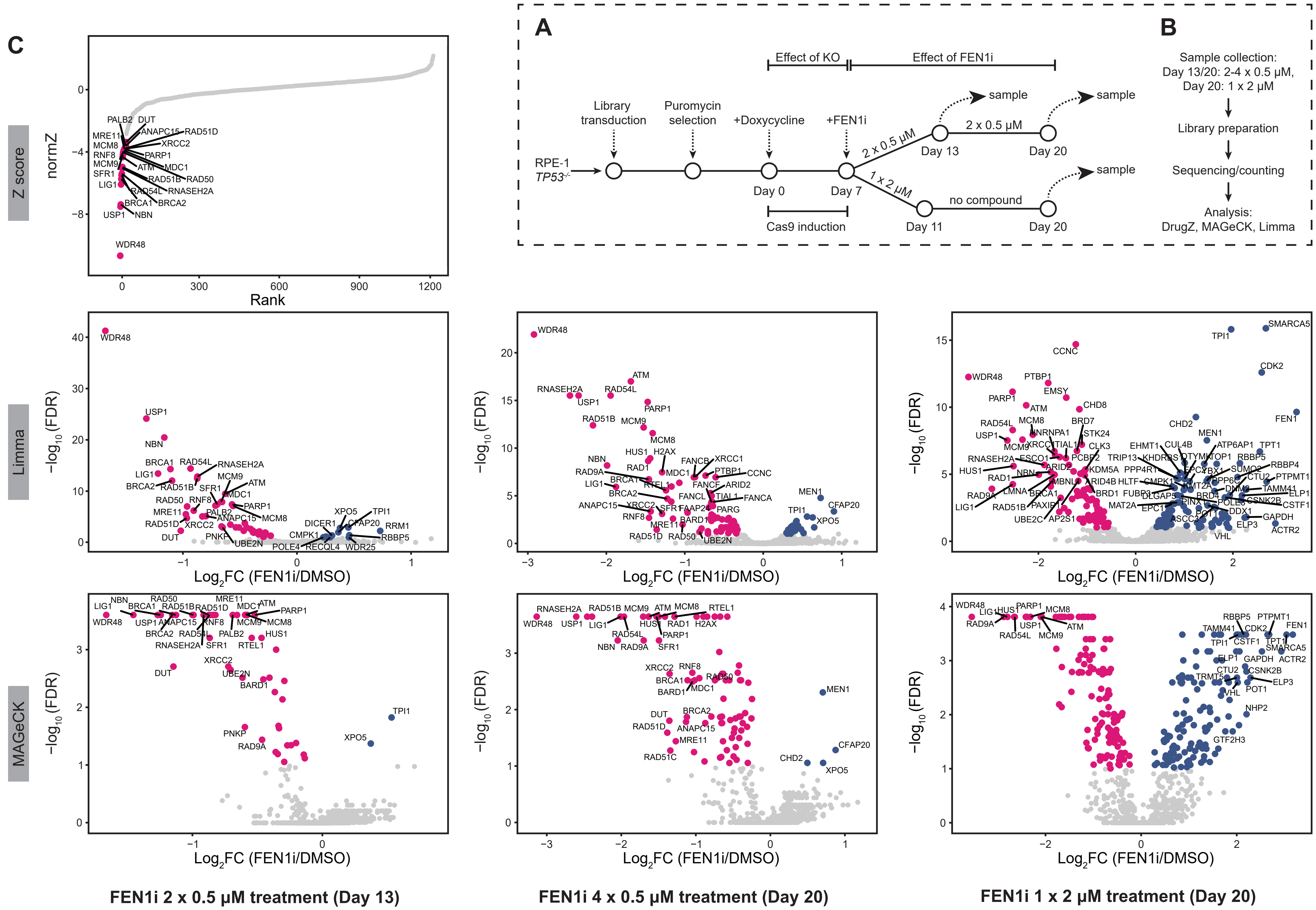

FEN1 - Supplementary Figure S7

Supplementary Figure 7. DDR-targeted CRISPR screens with MSC778 sampled with indicated doses at indicated timepoints using multiple analysis methods

A. Schematic of CRISPR screening strategy using a DDR-targeted CRISPR library in RPE-1 *TP53*$^{-/-}$ cells (Figure 7A-C) to identify genes whose depletion mediates sensitisation or resistance to MSC778.
B. Sample processing and analysis methods (DrugZ, MAGecK, Limma) used to assess output of CRISPR screens in (Figure 7A-C).
C. Plots showing genes targeted by sgRNAs that differentially dropped out or were enriched in MSC778 vs DMSO-treated RPE-1 cells in the CRISPR screen in RPE1 *TP53*$^{-/-}$ cells (Figure 7A-C). Samples were collected using schedule outlined in Supplementary Fig 7A and analysed using DrugZ, Limma or MAGeCK. Genes enriched or depleted with FDR < 0.1 and a Zscore of >3 or < -3 in DrugZ analyses are highlighted in magenta and blue, respectively. Genes enriched or depleted with FDR < 0.1 in Limma and MAGeCK analyses are highlighted in magenta and blue, respectively. Full guide counts are available in Supplementary Table 8.

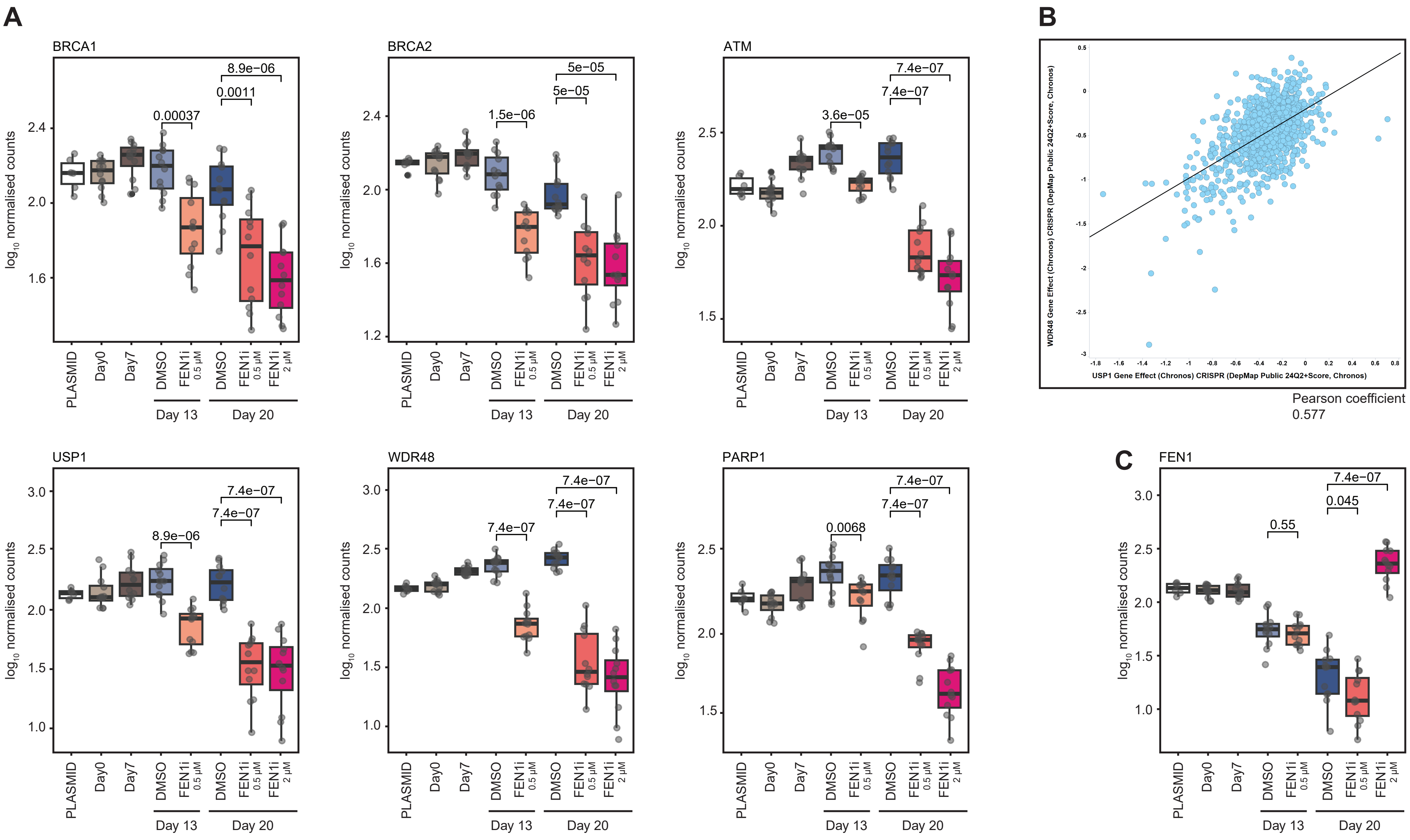

FEN1 - Supplementary Figure S8

Supplementary Figure 8. Examples of genetic interactions identified using DDR-targeted CRISPR library screens with MSC778

A. Box plots showing the depletion of composite guides targeting indicated genes.
B. Genetic dependencies of USP1 and WDR48 show a strong correlation in Depmap.
C. Box plot showing the enrichment of composite guides targeting FEN1 upon exposure to a high concentration of MSC778.

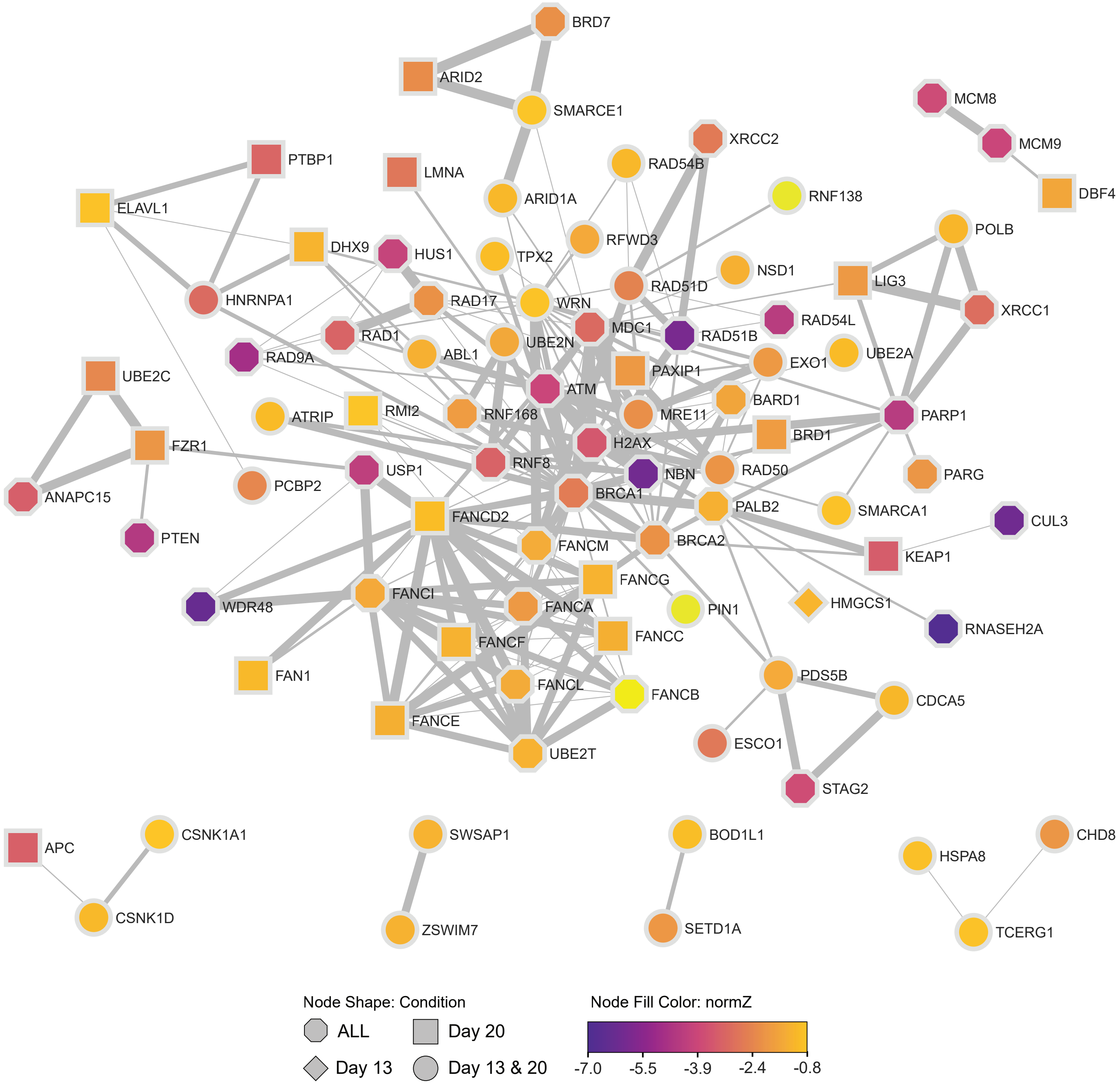

FEN1 - Supplementary Figure S9

Supplementary Figure 9. Expanded consensus of sensitisers to MSC778 identified using DDR-targeted library CRISPR screens

Protein-protein interaction network map of consensus sensitiser hits scoring in at least two of the three analysis methods.

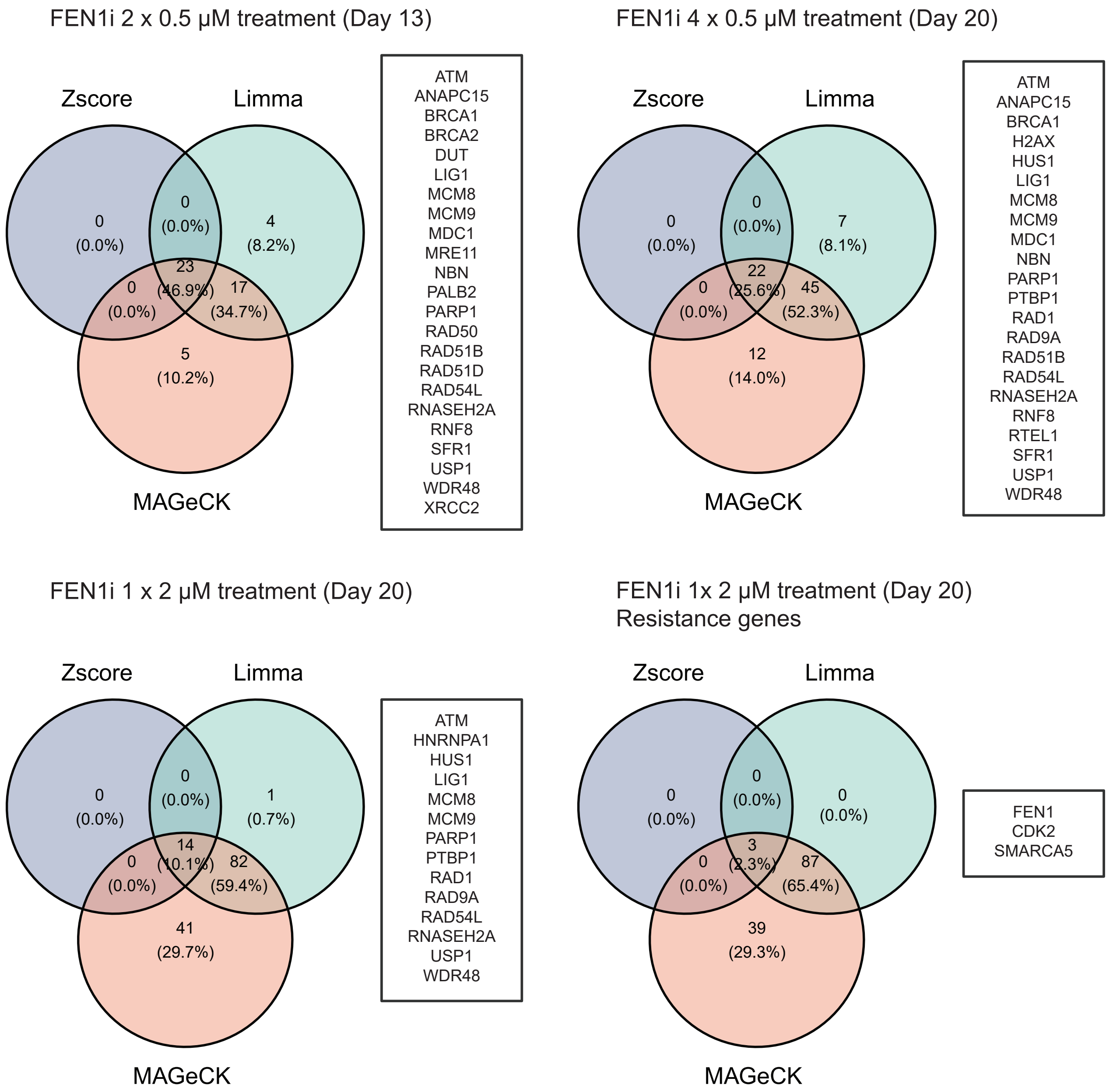

FEN1 - Supplementary Figure S10

Supplementary Figure 10. Overlap of hits scoring in DDR-targeted library CRISPR screens

Venn diagrams showing the consensus hits scoring in three analysis methods across indicated timepoints at indicated doses of MSC778.

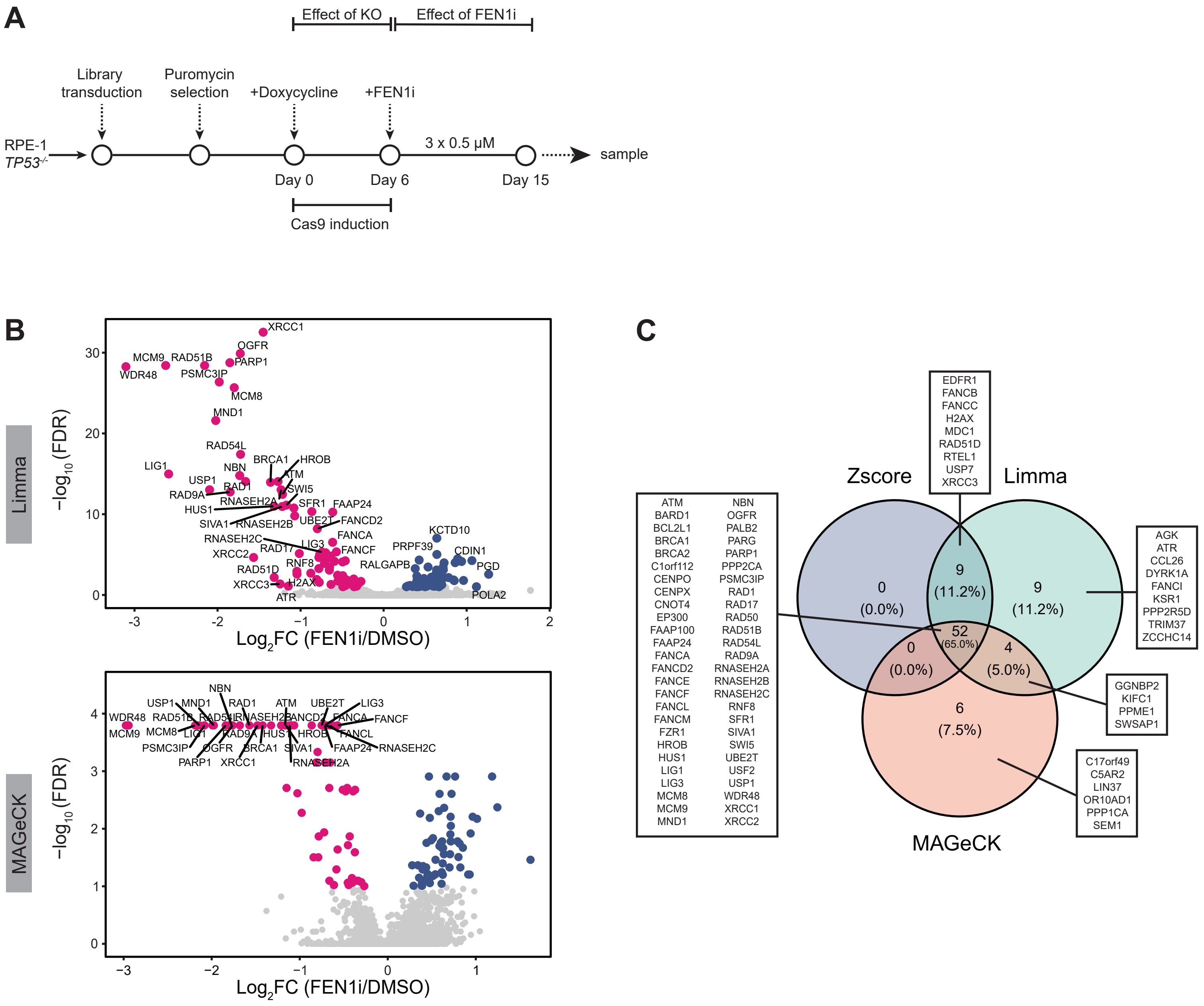

FEN1 - Supplementary Figure S11

Supplementary Figure 11. Whole genome CRISPR screen with MSC778

A. Schematic of CRISPR screening strategy using a whole genome CRISPR library in RPE-1 *TP53*$^{-/-}$ cells expressing a Scrambled gRNA (Figure 7D) to identify genes whose depletion mediates sensitisation to MSC778.
B. Plots showing genes targeted by sgRNAs that differentially dropped out or were enriched in MSC778 vs DMSO-treated RPE-1 cells in the whole genome CRISPR screen in RPE1 *TP53*$^{-/-}$ cells. Samples were collected and analysed using Limma or MAGeCK. Genes enriched or depleted with FDR < 0.1 are highlighted in magenta and blue, respectively. Full guide counts are available in Supplementary Table 9.
C. Venn diagram showing the consensus hits scoring in three analysis methods.

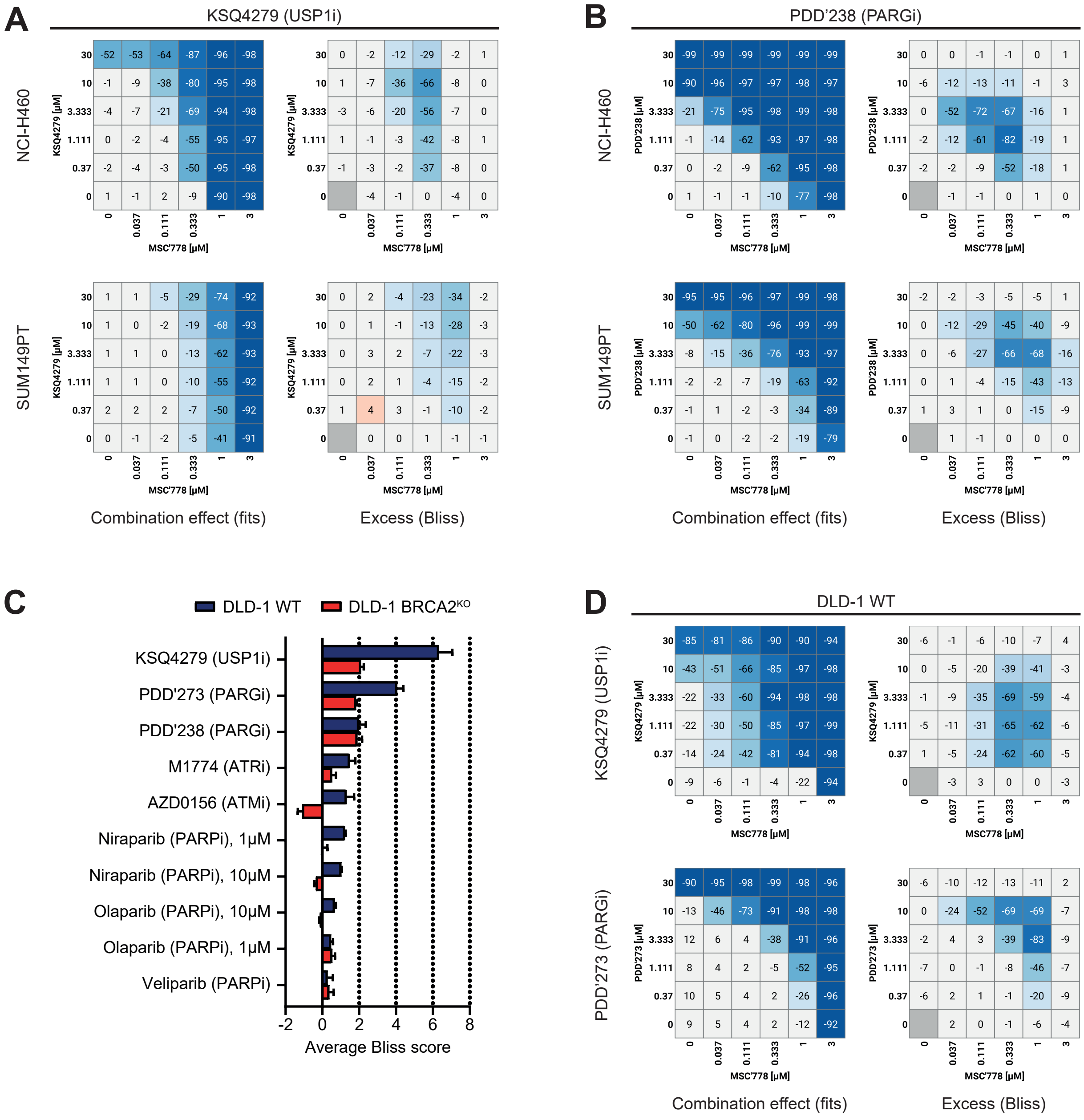

FEN1 - Supplementary Figure S12

Supplementary Figure 12. MSC778 synergises with inhibitors of USP1 and PARG in HRD and HRP cell lines

A. Visualisation of representative combination matrices of MSC778 and KSQ-4279 (USP1i) in NCI-H460 and SUM149PT cells showing effect on proliferation (left) and Bliss synergy (right).
B. Visualisation of representative combination matrices of MSC778 and PDD'238 (PARGi) in NCI-H460 and SUM149PT cells showing effect on proliferation (left) and Bliss synergy (right).
C. MSC778 combinations with indicated combination partner compounds in DLD-1 and DLD-1 BRCA2^{KO} cells. Bars represent the mean Bliss synergy score ± SEM of n $\geq$ 3 biological replicates, each performed in technical duplicates.
D. Visualisation of representative combination matrices of MSC778 and either KSQ-4279 (USP1i) or PARGi PDD'273 inhibitors in DLD-1 cells showing effect on proliferation (left) and Bliss synergy (right).

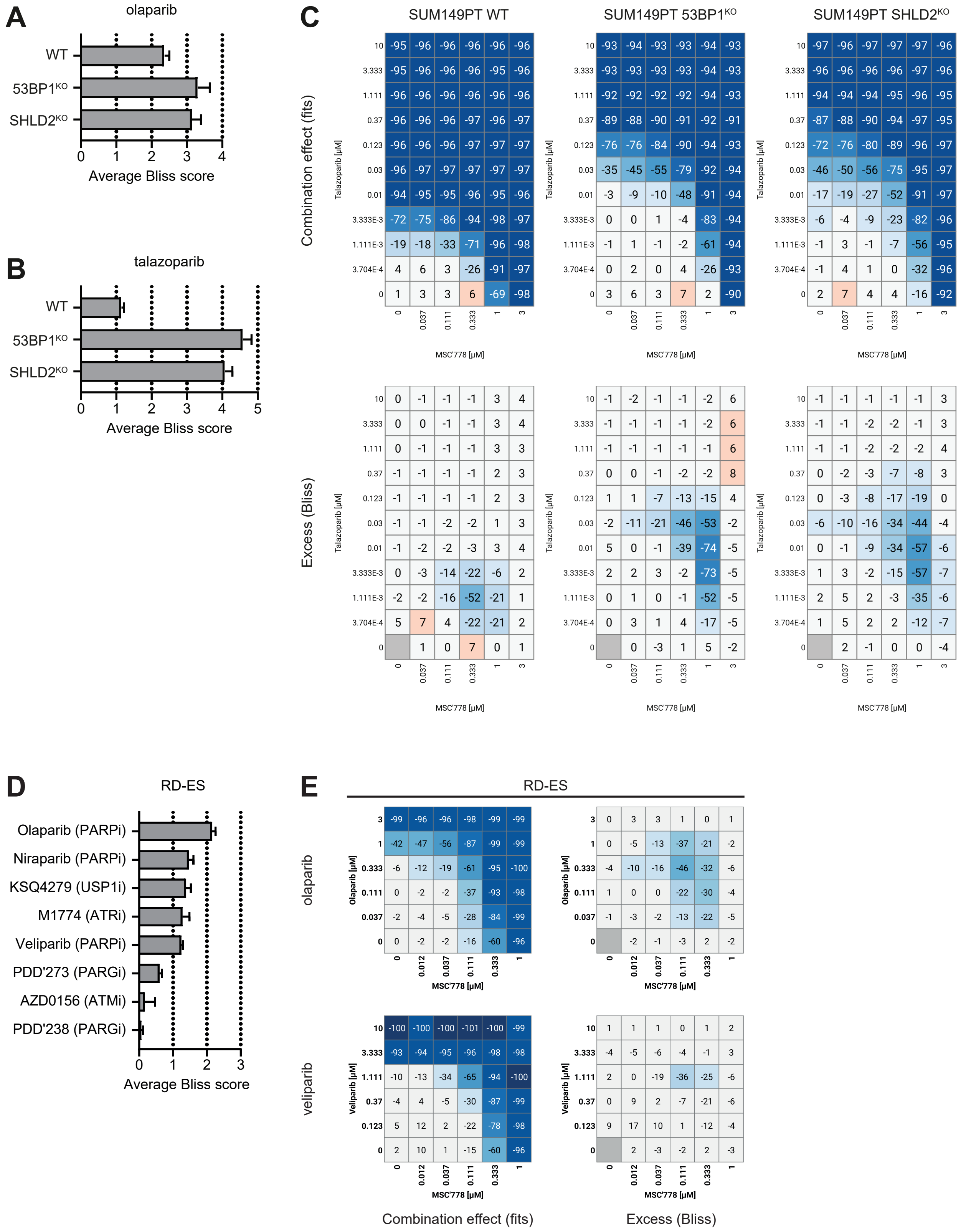

FEN1 - Supplementary Figure S13

Supplementary Figure 13. MSC778 synergises with PARP inhibitors in genetically engineered models of PARP resistance and RD-ES cells

A. MSC778 combinations with olaparib (PARPi) in the PARP inhibitor resistant SUM149PT 53BP1^{KO} and SHLD2^{KO} cell lines and their isogenic parental control (SUM149PT WT) cells. Bars represent the mean Bliss synergy score ± SEM of 3 biological replicates, each performed in technical duplicates.
B. MSC778 combinations with talazoparib (PARPi) in SUM149PT 53BP1^{KO} and SHLD2^{KO} cell lines and their isogenic parental control (SUM149PT WT) cells. Bars represent the mean Bliss synergy score ± SEM of 3 biological replicates, each performed in technical duplicates.
C. Visualisation of representative combination matrices of MSC778 and talazoparib (PARPi) in the PARP inhibitor resistant SUM149PT 53BP1^{KO} and SHLD2^{KO} cell lines and their isogenic parental control (SUM149PT WT) showing effect on proliferation (top) and Bliss synergy (bottom).
D. MSC778 combinations with indicated combination partner compounds in RD-ES cells. Bars represent the mean Bliss synergy score ± SEM of n $\geq$ 3 biological replicates, each performed in technical duplicates.
E. Visualisation of representative combination matrices of MSC778 and trapping (olaparib) and non-trapping (veliparib) PARP inhibitors in RD-ES cells showing effect on proliferation (left) and Bliss synergy (right).

SUPPLEMENTARY TABLES

Supplementary Table 1: Substrate oligonucleotides

Name	Target Assay	Sequence (5'-3')	Additional notes
Oligo 1	FEN1, GEN1	[FAM]-CGAGGGCATTCTAACAGTCATAGCG	
Oligo 2	FEN1	CTAAGTTCGTCAGGATTCCAC	
Oligo 3	FEN1, GEN1	CGCTATGACTGTTAGAATGCTGGAATCCTGACGAACTTAG	dT-18 modified with BHQ1
Oligo 4	GEN1	CTAAGTTCGTCAGGATTCCA	
Oligo 5	EXO1	CTAAGTTCGTCAGGATTCCACACAG-[FAM]	
Oligo 6	EXO1	CGCTATGACTGTTAGAATGCTTGGAATCCTGACGAACTTAG	dT-22 modified with BHQ1
Oligo 7	XPG	G*A*T*T*CAAGCAGTCCTAAGGTCGTAATAATGTCAGTATCC	dT-23 modified with BHQ1
Oligo 8	XPG	[FAM]-G*A*C*A*CACCTTAGGACTGCTTGAATC	
		* Denotes a phosphorothioate bond.	

Supplementary Table 2: Cell lines and culturing media

Cell line	Vendor	Catalogue #	Culturing media
DLD-1	Horizon Discovery	HD PAR-008	RPMI 1640 supplemented with 10% FBS
DLD-1 BRCA2^{KO}	Horizon Discovery	HD 105-007	RPMI 1640 supplemented with 10% FBS
HAP1 parental	Horizon Discovery	C631	IMDM supplemented with 10% FBS
HAP1 USP1 KO (5bp)	Horizon Discovery	HZGHC000620c012	IMDM supplemented with 10% FBS
HAP1 USP1 KO (13bp)	Horizon Discovery	HZGHC000455c007	IMDM supplemented with 10% FBS
HCT116	ATCC	CCL-247	McCoy’s supplemented with 10% FBS
HEK-293	ATCC	CRL-1573	MEM Eagle supplemented with 10% FBS
HeLa	ATCC	CCL-2	MEM Eagle supplemented with 10% FBS
HeLa SilenciX	Tebubio	01-00001	DMEM high glucose supplemented with 10% FBS and 250 µg/ml Hygromycin B
HeLa FEN1 SilenciX	Tebubio	01-00089	DMEM high glucose supplemented with 10% FBS and 250 µg/ml Hygromycin B
MDA-MB-436	ATCC	HTB-130	RPMI 1640 supplemented with 10% FBS
MG-63	ATCC	CRL-1427	MEM Eagle supplemented with 10% FBS
MHH-ES-1	DSMZ	ACC167	RPMI 1640 supplemented with 10% FBS
NCI-H460	ATCC	HTB-177	RPMI 1640 supplemented with 10% FBS
RD-ES-1	DSMZ	ACC260	RPMI 1640 supplemented with 15% FBS
SK-ES-1	ATCC	HTB-86	McCoy’s supplemented with 15% FBS
SUM149PT	BIOIVT	CS-07 (r03bt)	Ham's F-12 supplemented with 5% FBS, 10mM HEPES, 1 µg/ml Hydrocortisone and 5 µg/ml Insulin
SUM149PT crControl,(WT)	Described in PMID 36455556		Ham's F-12 supplemented with 5% FBS 10mM HEPES, 1 µg/ml Hydrocortisone and 5 µg/ml Insulin
SUM149PT crTP53BP1-2 KO			
SUM149PT crSHLD2-1 KO			
U-2 OS	ATCC	HTB-96	McCoy’s supplemented with 10% FBS

Supplementary Table 3: Compounds

Name	Target	Vendor	Catalogue #
talazoparib	PARP1	MedChemExpress	HY-16106A
olaparib	PARP1	SelleckChem	S1060
niraparib	PARP1	MedChemExpress	HY-10619
veliparib	PARP1	MedChemExpress	HY-10129
M1774	ATR	MedChemExpress	HY-111451
AZD0156	ATM	SelleckChem	S8375
KSQ4279	USP1	MedChemExpress	HY-145471
PDD00017238	PARG	MedChemExpress	HY-133530
PDD00017273	PARG	MedChemExpress	HY-108360
ART558	POLQ	Artios	n/a

Supplementary Table 4: Combination matrices

	Compound top dose [μM]										
**Cell line**	MSC'778	MSC'781	AZD0156	M1774	PDD'238	PDD'273	KSQ4279	olaparib	niraparib	talazoparib	veliparib
HCT116	3	3	10	0.3	n/a	n/a	30	n/a	n/a	n/a	n/a
MDA-MB-436	3	3	n/a	n/a	30	n/a	1	n/a	n/a	n/a	n/a
NCI-H460	3	3	10	n/a	30	n/a	30	10	n/a	n/a	n/a
SUM149PT	3	3	10	0.3	30	n/a	30	10	10	0.01	n/a
DLD-1 WT	3	3	10	0.3	30	30	30	10 / 1	10 / 1	n/a	3
DLD-1 BRCA2	3	3	10	0.3	30	30	30	10 / 1	10 / 1	n/a	3
SUM149PT WT	3	n/a	n/a	n/a	n/a	n/a	n/a	30	n/a	10	n/a
SUM149PT 53BP1^{KO}	3	n/a	n/a	n/a	n/a	n/a	n/a	30	n/a	10	n/a
SUM149PT SHLD2^{KO}	3	n/a	n/a	n/a	n/a	n/a	n/a	30	n/a	10	n/a
RD-ES	1	n/a	10	0.3	10	30	30	3	3	n/a	10

Supplementary Table 5: Antibodies

**Primary antibodies**					
**Target**	**Vendor**	**Catalogue #**	**Host**	**Dilution**	**Application**
ATR	Thermo Fisher Scientific	MA1-23158	Mouse	1:500	WB
ATR-pT1989	Cell Signaling	30632	Rabbit	1:1000	WB
BRCA1	Santa Cruz	sc-6954	Mouse	1:1000	WB
BRCA2	Millipore	OP95	Mouse	1:1000	WB
FANCD2	Novus Biologicals	NB100-182	Rabbit	1:1000	IF, WB
FEN1	Thermo Fisher Scientific	MA1-23228	Mouse	1:1000	WB
FLI1	Abcam	ab133485	Rabbit	1:1000	WB
GAPDH	Abcam	ab9485	Rabbit	1:1000	WB
H2AX	Cell Signaling Technologies	7631S	Rabbit	1:1000	WB
H2AX-pS139 (ɣH2AX)	Millipore	05-636	Mouse	1:2000, 1:1000	IF, WB
H3	Abcam	ab1791	Rabbit	1:2000	WB
KAP1-pS824	Abcam	ab243870	Rabbit	1:1000	IF
Mono-/Poly-ADP ribose	Cell Signaling Technologies	89190S	Rabbit	1:1000	IF
PARP1	Cell Signaling Technologies	9542S	Rabbit	1:1000	WB
PCNA	Cell Signaling Technologies	PC10 2586S	Mouse	1:1000	IF, WB
RAD18	Abcam	ab188235	Rabbit	1:1000	IF
RPA	Millipore	MABE285	Mouse	1:2000	WB
RPA-pS33	Cell Signaling Technologies	10148S	Rabbit	1:1000	WB
Schlafen 11	Abcam	ab271354	Rabbit	1:1000	WB
SP1	Cell Signaling Technologies	9389S	Rabbit	1:1000	WB
tRPA (9H8)	Abcam	ab2175	Mouse	1:1000	IF
Tubulin	Cell Signaling	86298	Mouse	1:2000	WB
Ubiquityl-PCNA	Cell Signaling Technologies	13439S	Rabbit	1:1000	WB
USP1	Bethyl Laboratories	A301-699A	Rabbit	1:1000	WB
Vinculin	Santa Cruz	sc-73614	Mouse	1:2000	WB

**Secondary antibodies**				
**Host/target species**	**Vendor**	**Catalogue #**	**Dilution**	**Application**
Goat anti-mouse Alexa Fluor 488	Invitrogen	A11001	1:2000	IF
Goat anti-rabbit Alexa Fluor 488	Invitrogen	A11034	1:2000	IF
Goat anti-mouse Alexa Fluor 568	Invitrogen	A11004	1:2000	IF
Goat anti-mouse Alexa Fluor 647	Invitrogen	A32728	1:2000	IF
IRDye 800CW Goat anti-Rabbit IgG	LiCor	926-32211	1:15000	WB
IRDye 680RD Goat anti-Mouse IgG	LiCor	926-68070	1:15000	WB
IRDye 800CW Goat anti-Mouse IgG	LiCor	926-32210	1:15000	WB
IRDye 680RD Goat anti-Rabbit IgG	LiCor	926-68071	1:15000	WB

Supplementary Table 6: Guide RNAs

Target	Vendor	Sequence 1	Sequence 2	Sequence 3
SCR	Synthego	GCACUACCAGAGCUAACUCA		
USP1	Synthego	AAAUUGCAAAGAAGAUUCUU	CAUUUCGGUUGAACAGCUCC	GGCGACUGCUUAACACACUG
WDR48	Synthego	CUUUGUUUGCAGUGCCUGUC	UAUACAUCUCUCUGGCCAA	UGUAUUCUGGUGGAAGGGAC

Supplementary Table 7: Software

Software	Version	Vendor
GraphPad Prism	10.2.3	GraphPad
Excel	2404	Microsoft
Adobe Illustrator	27.8	Adobe
Harmony	5.2	Revvity
Empiria Studio	3.0	LI-COR
Genedata Screener	21.0.1	Genedata
Protein Thermal Shift	1.4	Thermo Fisher Scientific
